# Mapping pure plastic strains against locally applied stress: Revealing toughening plasticity

**DOI:** 10.1126/sciadv.abo5735

**Published:** 2022-07-27

**Authors:** Thomas E. J. Edwards, Xavier Maeder, Johannes Ast, Luisa Berger, Johann Michler

**Affiliations:** Empa, Swiss Federal Laboratories for Materials Science and Technology, Laboratory for Mechanics of Materials and Nanostructures, Feuerwerkerstrasse 39, 3602 Thun, Switzerland.

## Abstract

The deformation of all materials can be separated into elastic and plastic parts. Measuring the purely plastic component is complex but crucial to fully characterize, understand, and engineer structural materials to “bend, not break.” Our approach has mapped this to answer the long-standing riddle in materials mechanics: The low toughness of body-centered cubic metals, where we advance an experimentally led mitigative theory. At a micromechanically loaded crack, we measured in situ the stress state applied locally on slip systems, and the dislocation content, and then correlatively compared with the occurrence—or not—of toughness-inducing local plasticity. We highlight limitations and potential misinterpretations of commonly used postmortem transmission electron imaging. This should enable better-informed design for beneficial plasticity and strength in crystalline and amorphous solids alike.

## Teaser

Correlative strain mapping reveals that toughness of microscale tungsten can be limited by crack tip dislocation nucleation.

## INTRODUCTION

How do crystal interfaces or second phase particles lead to increased strength upon plastic deformation? How can we promote the toughness of semi-brittle materials through crack tip plasticity? Fundamentally, this is asking: What determines whether, and at what rate, an applied stress will move a preexisting dislocation, activate a dislocation source in a crystal, or form a shear band in an amorphous material?

Physical models exist to describe these phenomena (*1*, *2*); however, these are based on a relatively small number of experimental observations as transmission electron microscopy (TEM) is time-consuming and limited to small volumes, as well as to the unavoidable plane stress state of thin foil specimens. As a result, mechanisms are often broken down into excessively simple relationships, which lose sight of the real complexity involved (*3*) and, over half a century later, remain hotly debated topics (*4*). To answer these questions experimentally, we need to be able to simultaneously measure, in a sample under load, both the local elastic strains (from which the stress state is deduced) and the pure plastic strains (to determine to what extent deformation mechanisms are activated, i.e., excluding the residual elastic contribution to common net elongation measurements of “plastic strain”) with sub–100-nm spatial resolution to resolve deformation features. No current method generally allows for this. TEM in situ mechanical testing may be used to measure pure plastic strains by counting the passage of individual dislocations, as recently proposed by Cui *et al.* (*5*) in the context of discrete dislocation models. However, not only is this technique laborious, requiring cryogenic conditions to limit spurious plasticity by atomic diffusion, but it is also not universally applicable: Amorphous materials, for example, do not deform by dislocation motion, nor do brittle ceramics above a certain size (*6*) or metals under diffusion creep.

**Fig. 1.**
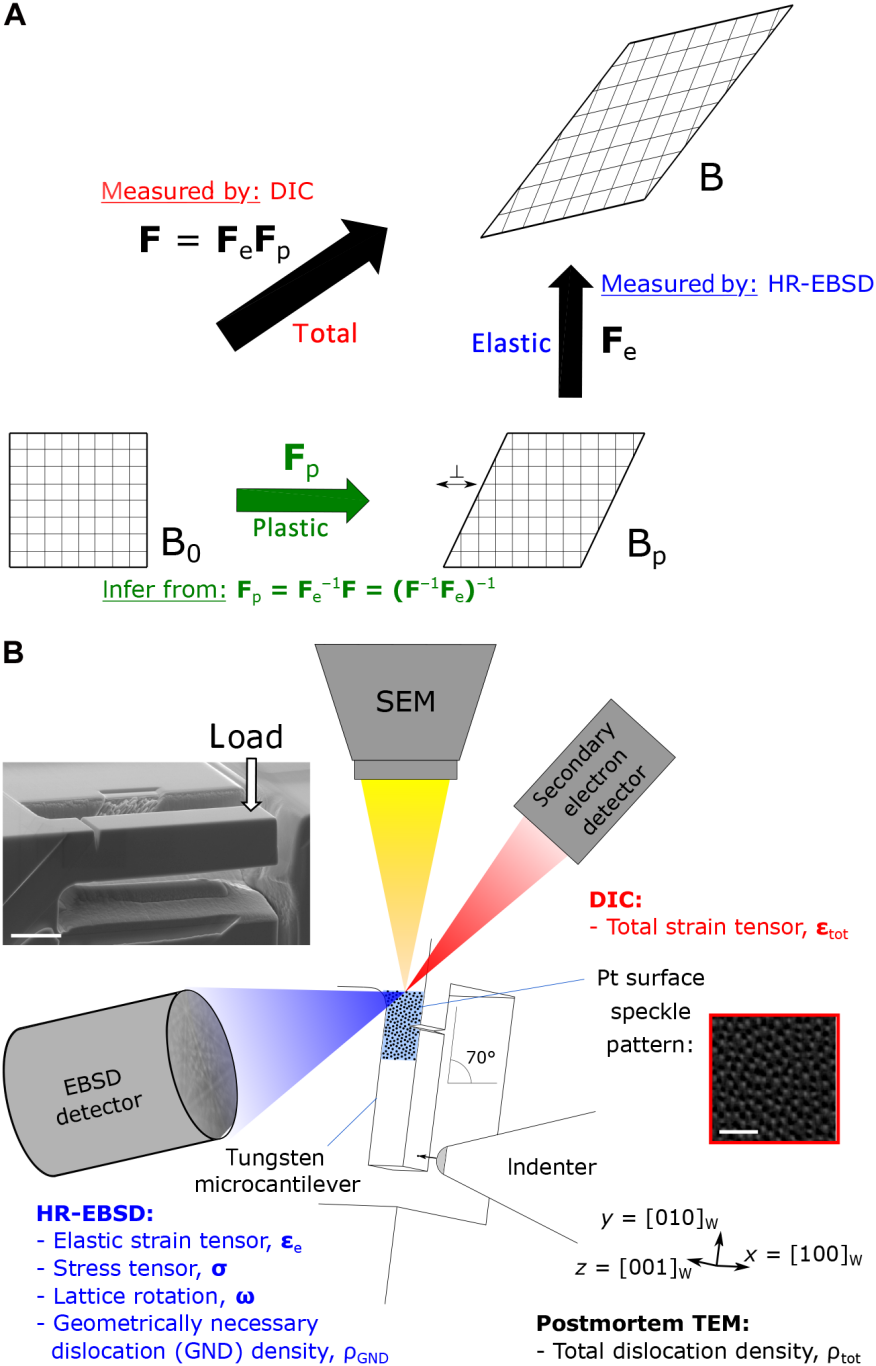
Elastic, plastic, and total strains measured at the notch tip during microcantilever testing by in situ diffraction mapping and speckle pattern imaging. (**A**) The total deformation gradient **F** measurable by digital image correlation (DIC) that maps an initial material configuration, B_0_, onto a final, B, may be decomposed (*73*) into a plastic deformation **F**_p_ (e.g., dislocation glide along crystal planes, indicated by inverted T symbol) to an intermediate configuration, B_p_, followed by elastorotational deformation **F**_e_ (stretching atomic bonds and rotating the crystal lattice; see text S1); the latter is measurable by HR-EBSD. The present study further extracts the purely plastic deformation through correlative measurement of the total and elastic deformation throughout mechanical loading. (**B**) Notched microcantilever bending of single-crystal pure tungsten was performed in a SEM with in situ correlative DIC and HR-EBSD strain mapping on a region of interest surrounding the notch. The scale bar on the inset secondary electron image of the larger of the two cantilever dimensions used is 5 μm long; the scale bar on the filtered (see Materials and Methods) secondary electron image of the Pt speckle pattern is 500 nm long.

To resolve this impasse, we propose a novel methodology: By measuring the total deformation and applying the reverse of the simultaneously measured elastic deformation (*7*, *8*), we can infer the pure plastic component (see Fig. 1A).

The measurement of elastic strain is generally performed using diffraction-based techniques for a wide range of material classes (see text S2 for a more complete review). Recently, techniques capable of mapping elastic strain and crystal lattice rotation with nanoscale spatial resolution have emerged, e.g., nanobeam synchrotron x-ray diffraction (*9*), scanning electron microscopy–based high-resolution electron backscatter diffraction (SEM HR-EBSD) (*10*), and four-dimensional scanning TEM (4D-STEM) (*11*, *12*).

Mapping of total strain with nanoscale resolution may currently only be performed using digital image correlation (DIC) methods, which track the movement of a pattern often imaged by SEM (*13*) and are therefore limited to measuring a 2D surface projection of the total deformation gradient. For further information on total strain mapping and the experimental complexity limiting extensive use of nanoscale DIC (nDIC) (*14*, *15*), see text S2.

The ability to simultaneously track elastic, plastic, and total strains throughout deformation, with the necessary sub–100-nm resolution (*15*), may be considered the holy grail in characterizing the micromechanisms of materials mechanics. This decomposition of strain would enable substantial broadening of our basic understanding of the elastic-plastic interplay, which is key to improving properties like the intrinsic toughness of materials. At a crack tip, stress must accumulate to activate plastic flow for blunting and shielding by energy dissipating permanent deformation, without the mechanical potential energy becoming high enough to cause excessive crack propagation (*16*). Furthermore, these measurements would be directly relatable to, and validate, model predictions of local elastoplasticity across the length scales, such as at the microstructural boundaries that often control the overall mechanical behavior. The separation of elastic and plastic strain components in the context of computational modeling, whether by continuum (*17*) or atomistic (*18*) approaches, is more commonplace as these values are calculated for each element or from atom positions at every computation step during deformation. Attempts have previously been made to measure both elastic and total strains either macroscopically (*19*), on distinct surfaces of a same test piece (*20*), or after unloading (*21*), requiring successive polishing steps, and local mapping is never achieved correlatively in the loaded state. This reflects the delicate balance that must be found here between the spatial and strain resolution of the two elastic and total strain mapping techniques to achieve measurements at regular time or strain intervals in the loaded state.

Here, we demonstrate this on a micrometer-scale sample under load, simultaneously measuring elastic and total strain fields at a notch tip with nanoscale resolution. The material studied, body-centered cubic (bcc) single-crystal tungsten, is semi-brittle at room temperature at the microscale (*22*). In itself, it is a material of particular interest for several applications, notably in nuclear fusion for its thermal exposure resilience as the divertor armor for International Thermonuclear Experimental Reactor (ITER) (*23*) and is an excellent illustration of a toughness-limited material lacking sufficient crack tip plasticity. Furthermore, the considerable prior knowledge for this notched microcantilever system (*22*, *24*, *25*) allowed the relatively low number of samples here, limited by the complexity of the experimental procedure, to be contextualized as a standard material response in crack growth strain energy release rate (see fig. S1) and compared with single-method studies [HR-EBSD (*22*, *25*, *26*) or low-resolution DIC (*27*)].

Hence, the present study does not simply set a benchmark in spatial resolution of strain mapping or demonstrate another incremental combination of techniques; rather, it enables a wealth of opportunities in materials development across a wide range of crystalline and noncrystalline materials unlocked by an understanding of the pure plastic component of deformation.

## RESULTS

**Fig. 2.**
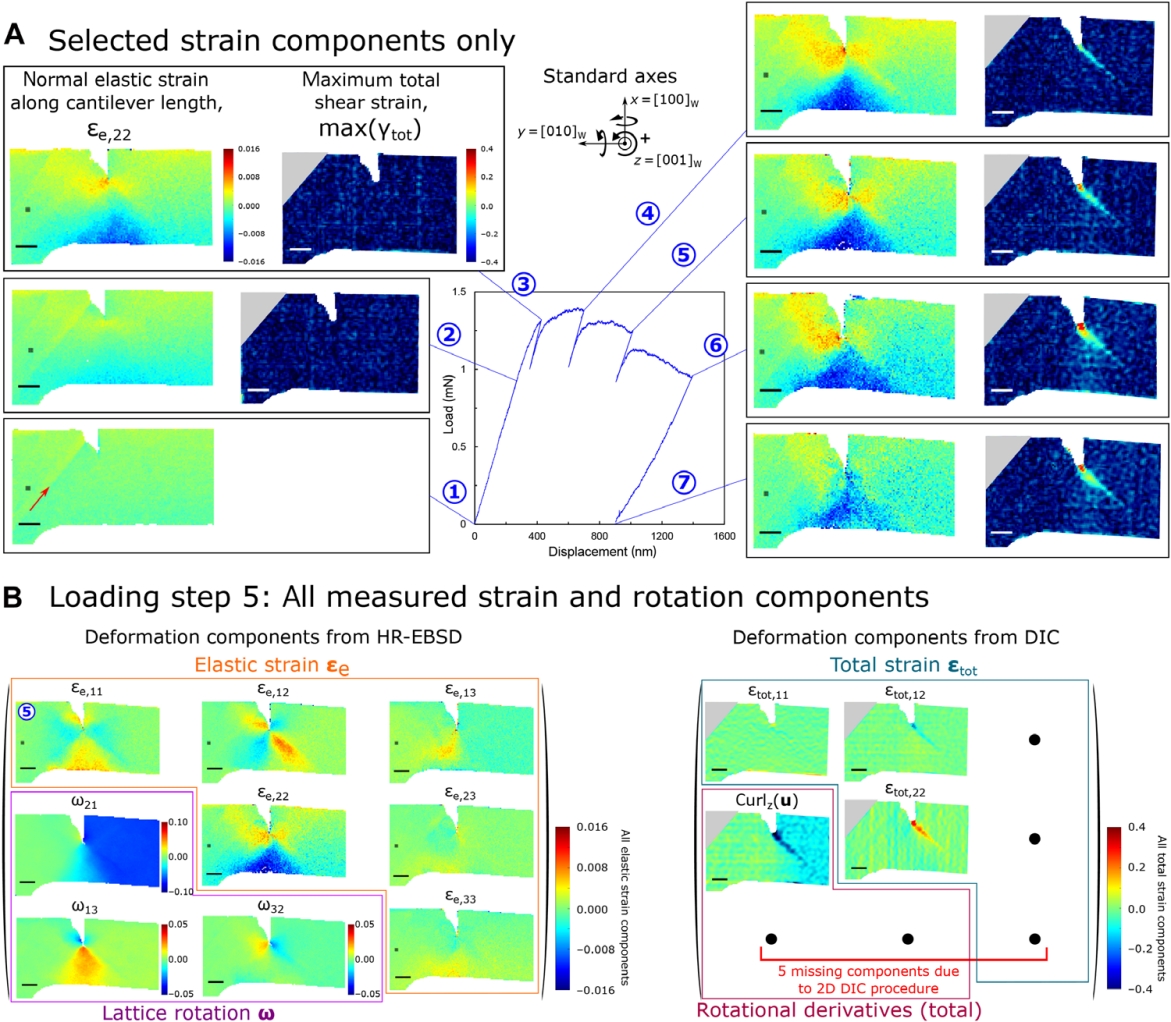
Compilation of elastic and total strain data for a 5μm-high microcantilever. - (**A**) The loading curve (applied force on cantilever arm end against the displacement of the spheroconical tip contact point) is overlaid with selected elastic (**ε**_e_) and total (**ε**_tot_) strain data. Indices 1, 2, and 3 correspond to components relative to the *x*, *y*, and *z* axes, respectively. Max(γ_tot_) is the maximum total shear strain, i.e., radius of the Mohr circle for the total strain state at a given location, and highlights the accumulation of shear-based plastic deformation. The Green-Lagrangian strain is plotted for total strain data to account for the potentially large strains (see text S1). The red arrow indicates a line artefact from FIB preparation of the notch. During the loaded hold periods, some elastic unloading is observed because of creep of the piezoelectic actuator (detailed further in Materials and Methods). (**B**) The complete elastic and total strain data measured are shown for loading step 5. The HR-EBSD and DIC strain and rotation data for all loading steps is given in figs. S3 and S4, respectively. The vectorial displacement field measured by DIC is **u**; hence, rotation components of total deformation may be expressed using curl(**u**). A right-hand rotation convention is used (see axes inset). The spatial resolution for elastic data is 75 nm; the total deformation was presently calculated with 16 × 16 px^2^ subsets with 25% overlap, yielding an 84.4-nm subset spacing. Errors of measurement for each deformation component are reported in full in Materials and Methods. In all maps, the scale bar is 1 μm long.

Pure tungsten focused ion beam (FIB) machined [001]-[010]-[100]–oriented cuboidal microcantilevers were loaded in situ in an SEM nanoindenter. The two cantilever sizes, referred to by their heights 5 and 3 μm, were FIB-notched for {100}<010> cracking sufficiently distant from the support of the cantilever to avoid elastoplastic interaction with the support material (*28*) and maintain symmetry locally about the notch (see fig. S2). Strain mapping (both elastic and total) was performed on the side surfaces of the cantilevers tilted at 70° to the horizontal before, at several intervals during, and after loading, as illustrated in Fig. 1B. See Materials and Methods for a complete description of the setup and the technical difficulties that were overcome to enable simultaneous nanoscale-resolution elastic and total strain mapping. In Fig. 2, the deviation of the loading curve from an elastic loading line at higher displacements, i.e., not perfectly brittle behavior, is characteristic of the limited plastic deformation observed in tungsten at room temperature at this scale (*24*) and requires the application of the *J*-integral method (*29*) to evaluate a conditional fracture toughness of 6.8 MPa m^1/2^ for the 5-μm notched cantilever.

### Loading until the elastic limit

**Fig. 3.**
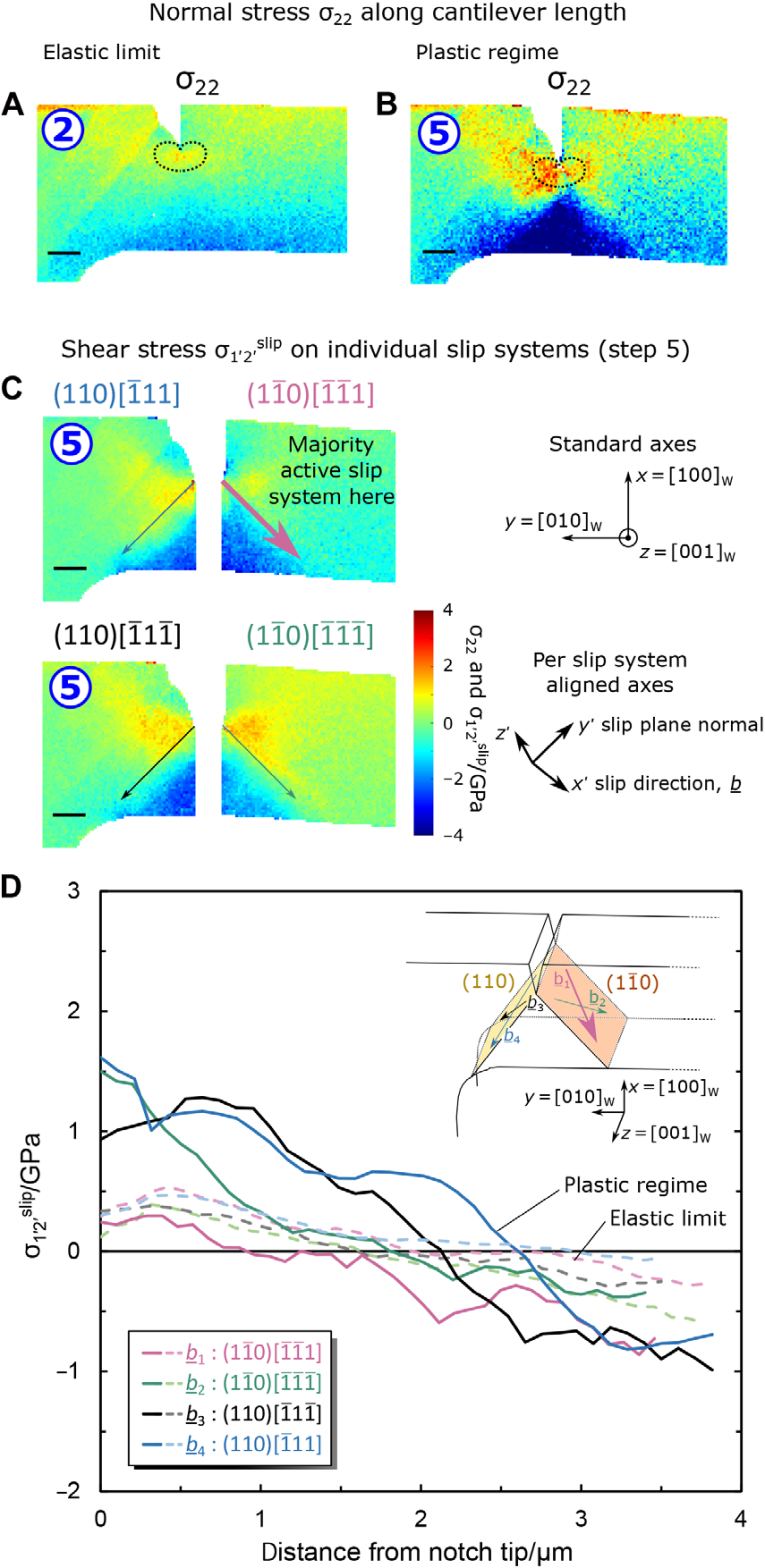
Normal and shear stress states at the cantilever surface. (**A** and **B**) Maps of normal stress along cantilever long axis, σ_22_, for elastic loading, step 2, and during crack growth, step 5, respectively, calculated from elastic strains measured by HR-EBSD in Fig. 2. The shape of the axial stress contours for a plane stress condition from linear elastic theory is overlaid at the notch tip, for reference; at step 5, asymmetry with higher crack opening stresses on the left of the notch is evident. As expected for cantilever bending, the lower half of the cantilever is in compression. (**C**) The shear stress resolved onto the plane and Burgers direction for the four $\{110\}<1\bar{1}1>$ slip systems diagrammatized in the inset in (**D**) (Burgers vectors, *b*_1_ to *b*_4_) is mapped, σ_1′2′_^slip^ (Peach-Koehler glide force for dislocations of edge or screw character). Disparate activating stresses at the cantilever surface in the plastic regime are observed in the profiles (solid lines), (D), along 45° lines emanating from the notch tip, which align with the (110) and $\left(1\bar{1}0\right)$ plane traces (corrected for lattice rotation to the right of the notch upon cantilever bending); at the elastic limit (dashed lines), the four slip systems were more equally loaded. Slip system 1, $\left(1\bar{1}0)[\bar{1}\bar{1}1\right]$, the most highly active of the four (see Fig. 5 and fig. S5), displays a much lower resolved shear stress during plasticity, suggesting from these data alone that either a lack of nucleation or excessive dislocation pinning is impeding the abundant plasticity on the other slip systems. σ_1′2′_^slip^ of all four systems is observed to change sign at a location defining the effective neutral axis of the notched cantilever, per slip system. A five neighboring point–centered averaging line is plotted here. Raw data points are in fig. S6, with error per data point. Scale bars, 1-μm long.

**Fig. 4.**
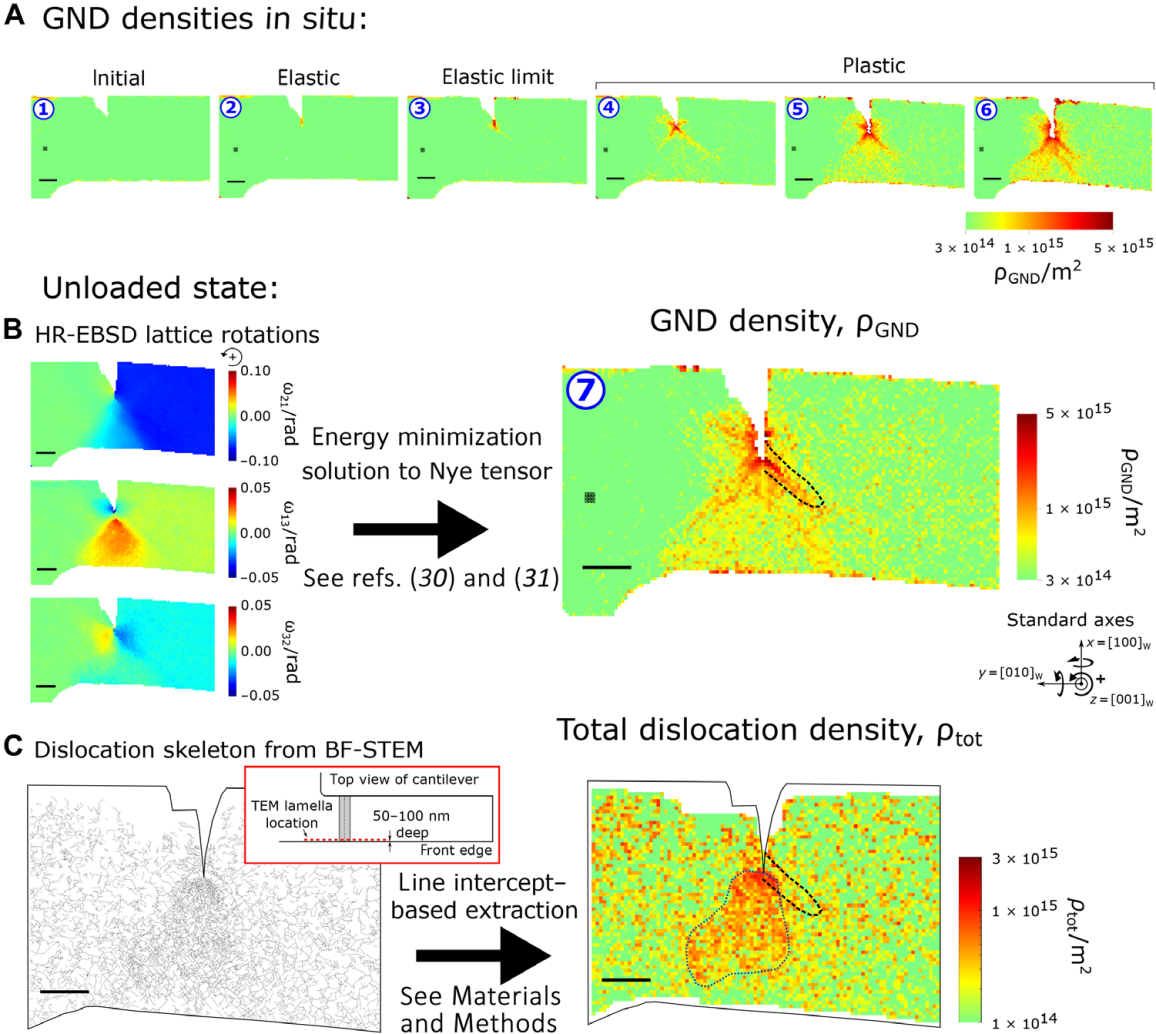
Comparison of total and geometrically necessary dislocation densities at the side surface of the 5-μm cantilever. (**A** and **B**) Maps of geometrically necessary dislocation densities, ρ_GND_, are derived from infinitesimal lattice rotations measured by HR-EBSD (Fig. 2) at successive loading steps. ρ_GND_ initially accumulates along two symmetrical 45° lines ahead of the notch tip, before a more uniform cloud of high ρ_GND_ develops ahead of the notch with further plastic loading and simultaneous stable crack growth. (**C**) Dislocation skeleton extracted from a postmortem bright-field scanning transmission electron micrograph (BF-STEM) of a lamella lifted just 50 to 100 nm deep from the cantilever strain mapped surface. A description of the dislocation structures observed is in text S4. Total dislocation density ρ_tot_ is extracted from the skeletonized dislocation array, with same spatial resolution as (B). Note differing color scales in (B) and (C); to understand this discrepancy, see text S5 and fig. S7, where noise levels are discussed. In contrast to symmetric ρ_GND_ after unloading in (B), ρ_tot_ in (C) is highest ahead of the notch to the center and left, outlined in dotted blue to guide the eye, while on the right, ρ_tot_ is no higher than in material much further from the notch, e.g., the cantilever root. This is exemplified by the black dashed region representing the band of high slip activity (see Fig. 5); here, ρ_tot_ remains low, while ρ_GND_ is above average for a given distance from the notch tip. In (**C**), ρ_tot_ shows greater statistical variations in low dislocation activity regions (far left and right on image) behind the notch than equivalent regions ahead of the notch due to lower foil thickness behind the notch (see Materials and Methods); average ρ_tot_ over larger areas, e.g., 10 × 10 px^2^, remains unchanged within error. Scale bars, 1 μm long.

The measurement of the elastic strain and infinitesimal rotations by HR-EBSD at the exposed side surface of a 5-μm microcantilever in the initial state (step 1, Fig. 2), at several positions in the loaded state (just below the elastic limit, 2; plasticity onset, 3; and elastoplastic, 4 to 6), and upon final unloading (*7*) enables derivation of local stress accumulation (Fig. 3) and the distribution of geometrically necessary dislocations (GNDs) [ρ_GND_, by energy minimization of the Nye tensor solution (*30*, *31*)] (Fig. 4, A and B). The ω_21_ rotation component straightforwardly indicates the progress of the beam bending procedure, including the retained net plastic deformation after unloading, while the ω_13_ and ω_32_ rotations indicate that spurious twist of the bulk of the beam around other axes is low (<0.5° relative to the root).

Initially upon loading (steps 2 and 3), localized tensile axial elastic strain ε_e,22_ and stress σ_22_ is observed to accumulate at the crack tip with the doubled-lobed geometry (Fig. 3A) expected from linear elastic fracture mechanics theory, while a compression region forms in the lower half of the cantilever. The corresponding Poisson contraction in other directions is also observed in these locations, e.g., ε_e,11_. Further description is given in text S3 for all loading steps.

The calculated GND densities (Fig. 4A) evidence a virtual absence of any plasticity; only a small increase is observed locally at the notch tip, which develops into the nucleus of a line of higher GND density emanating southeast from the notch tip at the elastic limit. By measuring the relative displacement of neighboring subsets of the optimized nanoscale Platinum (Pt) speckle pattern applied to the side surface of the cantilevers (nDIC; see Materials and Methods), the total strain at the cantilever side surface generated through combined elastoplastic deformation was mapped (Fig. 2). It is customary to report the maximum total shear strain max(γ_tot_) as it is a useful indicator of localized plasticity by essentially diffusionless deformation mechanisms, such as dislocation glide and twinning in crystals, and shear band formation in glasses, which are all shear based—unlike creep processes by boundary and bulk diffusion or by dislocation climb. At the elastic limit (step 2), in the loaded state, no total strain localization is measured by the nDIC method above the experimental noise threshold here. For a note on the apparent asymmetry of the notch shape in the strain maps, see text S6.

### Loading beyond the elastic limit

Through loading steps 4 to 6, progressively increasing plastic deformation and stable crack growth occur. The most unexpected, and perhaps most substantial, observation in the present correlative strain mapping study is seen in the max(γ_tot_) maps in Fig. 2A: A single band of high total deformation develops ahead of the notch tip, extending at a ~45° angle to the southeast. The shear stresses applied to the four.

The $\{110\}<1\bar{1}1>$ slip systems near the notch tip (Fig. 3, C and D) indicate that slip system 1, $\left(1\bar{1}0)[\bar{1}\bar{1}1\right]$, experiences the lowest activation stress of the four despite these slip systems being more equally loaded at the elastic limit. Atomic force microscopy (AFM) (text S7) and DIC strain mapping of this inclined surface (text S8) further reveal that slip system 1 is most abundantly active at the measured surface.

**Fig. 5.**
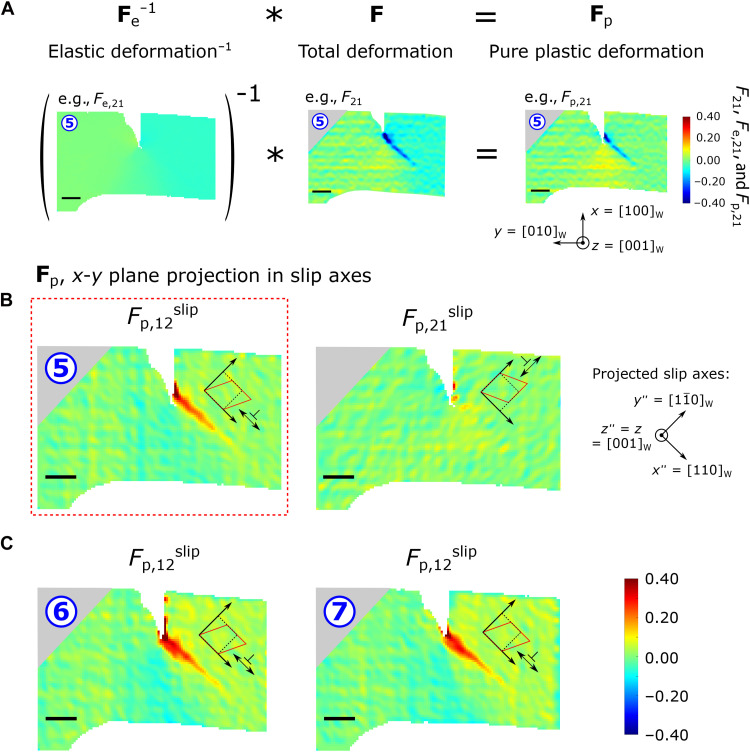
Extraction of the pure plastic component of deformation, F_p_. (**A**) Using the elastic deformation **F**_e_ data obtained by HR-EBSD and the total deformation **F#** data from DIC, Fig. 2, the pure plastic contribution to deformation **F**_p_ was calculated for loading step 5 here. The **F**_p_ components (dimensionless) are plotted on the deformed shape owing to a facilitation of the analysis procedure that this entailed (text S8). In (**B**) and (**C**), the simple shear components of the **F**_p_ tensor are resolved onto the surface projection of the slip systems identified in Fig. 3 due to the 2D limitation of DIC data. Insets indicate the type and positive sense of deformation per component (dotted black square deforms to red rectangle/rhombus). It is straightforward to note in (A) how deformation components in _e_ and **F#** such as lattice rotation cancel upon extraction of **F**_p_. Only the purely plastic crystal (for W here) deformation mechanisms, such as slip and cracking-mediated plasticity, remain. In *F*_p,12_^slip^, considerable slip plasticity is observed heading southeast of the notch tip, without any equivalent heading southwest in *F*_p,21_^slip^. The pitch of the square grid for **F**_p_ maps is 75 nm. Scale bars, 1 μm long.

Last, the pure plastic deformation gradient, **F**_p_, could be calculated (Fig. 5A), and, hence, the pure plastic strain, **ε**_p_, is extracted (see fig. S8 and text S8 for further details). However, in the context of dislocation-mediated plasticity, it is helpful to consider the simple shear strains (see shape change insets, Fig. 5B) revealed by the *F*_p,12_^slip^ and *F*_p,21_^slip^ components of **F**_p_, which are resolved in Fig. 5B onto the surface projection of the slip systems for loading step 5. These confirm high levels of shear plastic deformation emanating southeast of the notch, and none was measured to the southwest. The gradient of *F*_p,12_^slip^ plasticity along the slip line, negative traveling away from the notch, is suggestive of dislocation nucleation in the vicinity of the notch tip, followed by propagation of $\left(1\bar{1}0)[\bar{1}\bar{1}1\right]$ types outward from the tip, and eventual deceleration toward the soft σ_22_ and σ_1′2′_^slip,*b*1^ stress inversion boundary (Fig. 3) or exit off the side surface of the cantilever.

As plasticity ahead of the crack tip develops, so does a population of GNDs (Fig. 4A) congregating both within the first 500 nm of the notch tip and along bands extended at ~45° and ~60° downward from the notch tip. The distribution is symmetrical either side of the notch, with a slight skew to higher GND densities on the right-hand side, although differences are of the order of the noise threshold for the current correlative strain mapping setup.

Note that the symmetry of much of the elastic strain and lattice rotation–based HR-EBSD measurements under load substantially contrasts with the distribution of plastic strain. To understand the disparity between plastic strain and GND accumulation, further postmortem analysis techniques were undertaken.

### Unloaded state after deformation

After unloading (step 7), all stress components reduce notably; a reversal of total strain occurs immediately ahead of the initial notch tip as the crack partially closes and in the broader region of diffuse plasticity below the notch (Fig. 2). However, no reverse plasticity is observed along the slip line southeast of the notch tip (Fig. 5C). The densities of GNDs reduce throughout the region of interest (Fig. 4B); increased noise in the GND maps simply results from progressive accumulation of surface contaminants throughout the experiment.

A thin cross-sectional liftout of the crack region in Fig. 2 was produced by site-specific FIB preparation for transmission electron imaging. Care was taken that the lamella be from only 50 to 100 nm deep from the side surface of the cantilever; this was beyond the heavily FIB-damaged amorphous surface layer (*24*). Hence, the postmortem deformation structure imaged by TEM (Fig. 4C) reflected that of the strain-mapped surface insofar possible for effective contextualization of the strain mapping results relative to current standard TEM approaches. A description of the dislocation structures observed is given in text S4. The distribution of total dislocation density, ρ_tot_, was extracted from TEM images (Fig. 4C) using the same spatial resolution for binning as the GND mapping (see Materials and Methods) for direct comparison.

Figure 4B (ρ_GND_) and Fig. 4C (ρ_tot_) reveal considerable agreement in the general shape of the dislocation distribution: A high concentration within the first 500 nm of the notch tip then extends in specific directions below the notch, e.g., southwest. A noteworthy discrepancy however is the absence of a ~45° line extending southeast from the notch for ρ_tot_, as black dashed curves in Fig. 4 (B and C), i.e., specifically where the major line of plasticity is measured in *F*_p,12_^slip^ (Fig. 5B). Besides the general region of diffuse plasticity ahead of the notch tip also measured by DIC, there is therefore no correlation between a higher ρ_tot_ postmortem and lines of high plastic deformation during loading. Quite the reverse, one might term the line of heightened plasticity in this case a “mechanically annealed slip band,” where the successive passage of dislocations leaves a relatively obstacle-free path for further slip (see text S9). This is interesting in the context of the traditional cross-slipping screw dislocation mobility–controlled model for slip band formation in bcc metals, where immobile jogs from intersecting screw segments are heavily retained (*32*, *33*): Along this slip line, these have been almost entirely evacuated from the cantilever volume. One should also note the generally higher (factor of ~2) values measured for ρ_GND_ than ρ_tot_ despite the former being a subset of the latter; this previously observed (*25*) inconsistency is described and discussed further in text S5.

Unexplained discrepancies hence exist between the distributions of GNDs, total dislocations, stress, and plastic strain. For example, what causes an apparent annealed slip line to generate on one side of the notch and not on the other, and how does this asymmetry coexist with the symmetrical distribution of GNDs measured below the notch tip throughout loading, which are considered (*34*) to hinder dislocation motion by forest pinning?

## DISCUSSION

The correlative combination of elastic and total strain mapping during mechanical loading with nanoscale resolution undertaken here has enabled an unprecedented insight into elastoplastic deformation processes occurring at the tip of a propagated crack. The measurement of the evolving surface stress state along individual lines of high geometrically necessary dislocation content could be directly related to the existence, or not, of extensive dislocation glide–induced plasticity therein. In addition, although the ~16 μm by 12 μm area of interest is limited by the microcantilever geometries here, the present method could be applied directly to the study of much larger areas common of mesoscopic plasticity studies (*15*, *35*, *36*)—using the Focused electron beam induced deposition (FEBID) Pt method (see Materials and Methods), which operates here at a deposition rate of ~3 μm^2^ s^−1^ or, instead, an equivalently refined additive (*37*) or subtractive (*36*) bulk-processing pattern.

Further application of nanoscale resolution combined elastic and total strain mapping to in situ TEM deformation studies is similarly envisaged to further increase spatial resolution. Hence, not only dislocation-mediated plastic deformation but also diffusion-controlled creep at grain boundaries and in the bulk of nanocrystalline grains, or amorphous shear-banding, could be measured during loading by DIC of appropriately sized nanoparticle surface markers (*38*, *39*). The corresponding maps of elastic strains evolving during loading could again be measured by pointwise collection of diffraction patterns according to one of several existing techniques (*11*, *12*, *40*).

The differences here between the elastic shear stress applied to individual slip systems, GND densities, and plastic strain fields reflect the reality that high stresses do not straightforwardly lead to plastic deformation and GND accumulation. Similarly, defect structures imaged postmortem by TEM are poor indicators of the stress and plastic strain distributions, leading to them prompting a warning against overinterpretation, much as Kuhlmann-Wilsdorf (*41*) evoked on the subject of dislocation structures being deceptive: “Often there is no recognizable connection between the surface markings and the underlying dislocation structures, [which] are the grave yards of previously vigorous dislocations and, like human grave yards, do not give much information on preceding travels, whereas slip lines do”. One might say that the loss at the cantilever surface of those dislocations inducing the most intense plasticity here is rather a case of “ashes to the wind”: The conditions of material constraint by microstructural boundaries, or conversely free surfaces, cannot be overlooked. The analysis here of pure plastic shear *F*_p,12_^slip^ (Fig. 5 and fig. S10) can specify the number of dislocations that necessarily glided along the southeast slip plane at each loading step—up to 7 × 10^2^ near the notch after unloading—despite some being part of the “lost population.” Side-surface slip steps in previous equivalent studies (*22*, *24*) indicate that also symmetrical $\{110\}<1\bar{1}1>$ slip lines can instead develop below the notch tip; similarly, dislocation depletion regions may be reversed about the notch relative to Fig. 4: The occurrence appears stochastic. This highlights the need to understand plasticity on a case-by-case basis, with complete elastic and plastic strain mapping, as well as additional information by postmortem TEM.

A finite element (FE) formulation of mobile dislocation density (*17*), ρ_m_, is useful to analyze the results and is expanded upon in text S10, where further commentary on limitations to the crystal plasticity FE (CPFE) method raised by the present study is given. One may infer with such a formulation for dislocations that the favorable experimentally determined conditions to generate high dislocation-mediated plastic strain at a {100}<010> notch tip (i.e., effective blunting and toughening) in tungsten at room temperature are a lower density of mobile dislocations yet that move with a high velocity—for this, 5-μm sample scale and at the present loading rate (text S11 discusses the impact of load pauses for map acquisitions). This is independent of the local ρ_GND_: GND accumulation does not necessarily indicate abundant plasticity. This is relevant to the left-hand side of the notch, where the higher density of mobile dislocations is understood to move considerably more slowly, with the net shear rate $\overset{\cdot }{\gamma }$ at least an order of magnitude lower than on the right—based on the plastic shears achieved there (Fig. 5B). This suggests that effective plastic deformation for toughening requires an exuberant source of fast moving dislocations. However, in CPFE models (*34*, *42*), the nucleation rate and spatial distribution thereof, as well as the possible surface loss starvation of mobile dislocations, are not considered. Although discrete dislocation models may partially consider this, the variable rate of activation of the source is incompletely considered (*28*), being invariably related to the applied shear stress (*43*), which is seen here to not effectively describe the local abundance of plasticity-mediating dislocations.

It is currently thought that the brittle-ductile transition (BDT) of tungsten is dislocation mobility–controlled, i.e., not nucleation limited (*44*, *45*); this is reviewed further in text S12. Here, the temperature-loading rate parameters probe the material in the semi-brittle regime at the upper end of this transition (*22*). It is apparent from the results of the present study that in this latter regime, for this sample size, the controlling mechanism differs from that thought to operate at the BDT: At all stages during loading, the GND distribution that may serve to forest-limit dislocation mobility remains symmetrical about the notch, unlike the zones of major plasticity. A complete theory should relate the measured stress states to the occurrence, or not, of plasticity. It may seem intuitive that the higher crack opening stress σ_22_ that develops on the low-plasticity left side of the notch should be avoided, but why then do higher slip system–resolved shear stresses on the left side (Fig. 3) not lead to equivalently active slip lines? That is, does the applied shear stress not control dislocation velocity or the activation rate of individual sources? Understanding this would undoubtedly improve our fundamental mechanistic models for plasticity and lead to the design of tougher, more crack-tolerant engineering materials by the promotion of beneficial crack tip plasticity in this regime. Hence, the limitation of room temperature toughness of W by source starvation should be considered at this size scale and this loading rate despite the considerable density of crack tip dislocation tangles in postmortem TEM (fig. S11).

How might tungsten be modified to enhance dislocation nucleation at the notch tip? Previous attempts (*45*) at increasing dislocation source density in tungsten through cold working led to a BDT increase of 100 K related to premature forest hardening. Instead, a solution to mitigate this nucleation limit may lie in recent 3D molecular dynamics simulations: Reorientation of curved crack fronts in bcc metals (*46*) away from {100}<010> promotes fine-scale twinning, absent here, which acts as an emission source for the desired blunting and shielding dislocations. This reorientation may be achieved by intersection of the propagating crack with nanovoids (*47*) and specific dislocation types (*48*)—a process that itself promotes further rapid dislocation multiplication. Although experimentally verified in room temperature quasi-static conditions, twinning—and anti-twinning—in tungsten has only been observed at the very high stress states achievable in 20- to 40-nm test piece sizes (*49*, *50*). Formation of the intermediary nanotwins at the crack tip at this microscale may be facilitated more simply by alloying to reduce the stacking fault energy, which only Mo, Re, and Os are known to achieve, among a short list of elements evaluated thus far at 2 atomic % by first principles calculations on ${\left\{11\bar{2}\right\}}_{W}$ planes along the twinning <111> directions (*51*). Alternatively, alloying could be directly used to reduce the dislocation nucleation barrier as was recently achieved on a face-centered cubic system (*52*). The correlative strain mapping method presented here is capable of directly evaluating the success of these modifications.

A final thought concerns the effect of sample size: Previous studies found that smaller test piece dimensions led to lower fracture toughness, predicted to result from a more geometrically limited plastic zone (*24*, *53*). Strain mapping of a reduced cantilever size is related in text S13 and confirms this. With smaller sizes, plastic strain is more limited spatially and in intensity yet remains asymmetric about the notch like the larger cantilevers. Solutions to toughen the larger cantilevers by amplifying dislocation multiplication at the crack tip should also improve size-limited toughness by increasing the intensity of plasticity, and potentially notch-tip GND accumulation, preceding fast fracture.

We have therefore introduced a new approach to the study of the deformation of solids that generates a complete description of the elastic and plastic processes occurring at the nano and microscales relevant to their microstructure. Although applied in the current study to a crystalline material, the method could straightforwardly be adapted to the investigations of other classes of solids, e.g., glasses or polymers, by using alternative in-SEM scattering techniques (*54*–*56*) detecting transmitted electrons rather than the backscattered here.

This has enabled us to determine that toughening plasticity in tungsten in small-scale mechanics must be limited in the present supra-BDT conditions by an insufficient density of dislocation nucleation sources capable of producing an abundant population of highly mobile dislocations despite measurably ample stresses being applied locally. The lack of mobility-limiting dislocation array accumulation where extensive plasticity occurs here, which would normally occur at the BDT, illustrates the clear mechanistic distinctions accessible with this novel methodology.

The opportunities for the application of this approach to existing problems in mechanical metallurgy are vast. A first example here is fracture mechanics, be it plasticity-induced quasi-static toughening or cyclic processes at a fatigued crack tip—of particular current interest in the context of emerging lightweight semi-brittle intermetallics (*57*) not only for energy and transport applications but also for novel engineering ceramics (*58*) or quasi-plastic biocomposites (*59*). Further interest is expected in the field of strengthening of materials to understand the exact mechanisms by which the introduction of boundaries and second phase particles—crystalline or amorphous, harder or semipermeable to defects, precipitates or insoluble—results in strength. The present approach is competent to resolve and provide data directly comparable to model predictions (*60*) for the relationship between local stress accumulation and eventual plastic deformation in that location, which current physics-based computational models aim to capture from the atomic (*61*) to the grain scale (*62*, *63*).

## MATERIALS AND METHODS

### Material and metallographic preparation

Microcantilever test pieces were machined from a (100)-oriented pure W single crystal by Ga^+^ FIB machining in a dual-beam FIB-SEM (Lyra, Tescan, Czech Republic) (see fig. S2). The side faces of the cantilevers were tilt compensation–milled to align with the (010) and (001) planes to within less than 1°, from currents of ~10 down to ~2 nA at 30 kV, according to a method established by the authors in previous publications (*22*, *25*), which also covers initial preparation of the W sample. Notching of the cantilevers was achieved using a 300-pA FIB current, as in (*25*). A summary of the sample dimensions for the two cases to evaluate the effect of sample size on the elastic and total strains generated on the cantilever side surface is given in table S1. For both cases, three identical test pieces were prepared and tested in differing combinations of correlative DIC + HR-EBSD and DIC only; HR-EBSD—only studies were the subject of previous work (*25*).

### Mechanical testing

Micromechanical testing was undertaken with a 1-μm radius conospherical diamond tip (Synton-MDP AG, Switzerland) at room temperature using an Alemnis nanoindenter (Alemnis AG, Switzerland) in situ in an SEM (Lyra, Tescan, Czech Republic). Initial contact with the cantilevers for punch alignment and contact surface determination did not exceed loads of 50 μN. Microcantilever bending tests were carried out in displacement-controlled mode at a displacement rate of 5 nm s^−1^ (giving effective stress intensity factor rates of the initial linear elastic segment for the larger notched cantilevers of 0.05 MPa·m^1/2^ s^−1^ and 0.06 MPa·m^1/2^ s^−1^ for the smaller). An additional 40-Hz sinusoidal, 5-nm amplitude oscillation was superimposed on linear loading to measure the contact stiffness, as previously performed (*22*). Deformation was paused at several points during loading to acquire HR-EBSD (lasting 18.1 min for 5-μm high cantilever) and DIC (2.7 min) strain maps.

Mechanical test data were analyzed according to the *J*-integral method (*29*), which accounts for the partial ductility of crack growth at room temperature in micromechanically tested tungsten (*24*) and yields a conditional mode I fracture toughness *K*_Iq_. On the basis of a 50- to 100-nm SEM imaging error of measurement of the point of contact of the spheroconical tip, an error analysis for the *J*-integral calculation yields ~0.5 MPa m^1/2^ for the error in *K*_Iq_.

### Mapping of surface strain

#### *HR-EBSD elastic strain mapping*

The sample was mounted on the in situ loading rig in the same orientation as in previous work (*26*) to enable the acquisition of Kikuchi patterns from the 70° (to the laboratory horizontal) exposed side faces of the microcantilevers using a DigiView camera (EDAX, NJ, USA) at regular intervals during the mechanical tests, with the specimen still under load, using 20-kV electron acceleration and a current of 16 nA. EBSD scans were performed with a 75-nm step size, 100-ms dwell, and 2 × 2 px^2^ binning of the Kikuchi patterns. The elastic strain and stress as well as the infinitesimal lattice rotation tensors were extracted using the HR-EBSD technique developed by Wilkinson *et al.* (*64*), implemented in CrossCourt 4 (BLGVantage, UK). A lower bound (noise threshold) for the measurable GND density was further estimated for tungsten, as previously discussed (*25*): 5.9 ± 2.2 × 10^13^ m^−2^ with the applied surface Pt speckle pattern at loading step 1 (Fig. 2). Reference patterns for elastic strain mapping were selected in the cantilever root, far from the notch tip elastic stress field, as a 3 × 3 kernel (2 × 2 for 3-μm cantilevers) from which the pointwise lowest noise correlation was selected; these are indicated throughout as gray squares on the HR-EBSD datasets. Filtering of patterns by image quality before correlation (>1.5 × 10^4^ retained) and by a mean angular error threshold (<0.003) afterward was performed. Noise levels for elastic strain, stress, and lattice rotation mapping were determined from the SD of HR-EBSD measurements on the cantilever in the undeformed state as: 3.4 × 10^−4^ for ε_e,11_, 2.6 × 10^−4^ for ε_e,12_, 6.4 × 10^−4^ for ε_e,22_, 3.0 × 10^−4^ for ε_e,31_, 1.9 × 10^−4^ for ε_e,23_, 3.4 × 10^−4^ for ε_e,33_, 0.20 GPa for σ_11_, 0.08 GPa for σ_12_, 0.30 GPa for σ_22_, 0.10 GPa for σ_31_, 0.06 GPa for σ_23_, 6.4 × 10^−4^ for ω_12_, 5.4 × 10^−4^ for ω_31_, and 5.7 × 10^−4^ for ω_23_.

### n-DIC total strain mapping

Surface strain mapping of the exposed side face of the microcantilever was achieved by DIC of a Pt speckle pattern as described below. The Pt pattern was imaged by secondary electron imaging (20 kV, 800 pA, 100 μs px^−1^ dwell) in situ during testing at regular intervals, before the EBSD pattern acquisition at each step, with the imaged surface still at 70° tilt, such that a software-based geometric adjustment of the SEM raster and dynamic focus variation was required to compensate the tilt. Pixel sizes were 7.05 nm px^−1^ for the 5-μm cantilevers and 4.70 nm px^−1^ for the 3 μm ones. The cumulative correlation of the image series relative to the undeformed state was performed using commercially available DIC software (DaVis, LaVision, Germany) with 25% subset overlap. Subset sizes differed in pixels between cantilever geometries due to differing region-of-interest sizes; hence, strain mapping resolution is reported on a case-per-case basis in the corresponding figure caption. Before correlation, images were bandwidth-filtered. DIC of repeatedly imaged regions yielded a noise level (SD) in ε_tot,11_ of 0.023, ε_tot,12_ of 0.017, ε_tot,22_ of 0.031, max(γ_tot_) of 0.013, and curl_z_(**u**) of 0.041 rad (Gy) for the large-notched microcantilever in Fig. 2 at the same DIC resolution. The repeated images were acquired at maximum cantilever deformation, yielding a conservative, worst-case noise measurement. For the small cantilever in fig. S9, these strain errors were equivalently 0.009, 0.010, 0.030, 0.011, and 0.020 Gy, respectively. It should be emphasized that this noise level results not only from both SEM scanning imperfections but also from the poor electron yield in the detector direction at the high specimen tilt angle required for HR-EBSD mapping and is considerably higher than otherwise achievable in a dedicated SEM-DIC setup (*65*). Although this noise level prohibits the capture of elastic strain, it is nevertheless well suited to measure the high strains of plastic flow. DIC strain maps are mapped onto the deformed geometry specific to each loading step; as strains may be large, the Green-Lagrangian strain is plotted (see the Text S1). Rotational derivatives may be calculated according to expressions in (*15*).

### Speckle patterning

Achieving a speckle pattern suitable for DIC strain mapping at the high resolutions accessible by SEM imaging (*8*, *13*, *37*) but nevertheless sufficiently electron transparent to allow acquisition of sufficiently clear Kikuchi patterns for HR-EBSD strain mapping was crucial for the success of the present study. A preliminary study was performed on W to determine optimal conditions for an electron beam (e-beam)–deposited (FEBID) Pt gas injection system pattern (Helios NanoLab 660i, FEI, USA). The size of the speckles, their spacing, the dwell time of the e-beam per point, and the type of Pt-gas injection system (GIS) system used—single Pt precursor needle or multiprecursor needle (FEI, USA)—were all varied; the e-beam conditions themselves had been optimized in a previous study (*66*). From the subset of speckle patterns that yielded a sufficient imaging contrast to be suitable for SEM-DIC strain mapping, the pattern that produced the highest image quality index (OIM Analysis, EDAX, USA) for the Kikuchi patterns of W was selected. These deposition conditions are given in table S2. Speckle pattern optimization for combined HR-EBSD and nDIC over a wider range of materials will be discussed in detail in a future publication.

### Manipulation of strain and stress data

Rotation of elastic strain and stress data for alignment with slip directions, as well as the combination of total and elastorotational strain mapping to extract the pure plastic deformation gradient tensor components, was performed using functions written in MATLAB (MathWorks, UK). Errors for the calculated **F**_p_ components (see fig. S12) were determined by error propagation for the formula in the Text S8, noting care required for propagation upon matrix inversion.

### Transmission electron imaging

Thin lamella liftouts of the W cantilever test pieces for further analysis requiring electron transparency were obtained by a Ga^+^ FIB liftout process (Helios NanoLab 660i, FEI, USA) with a preliminary protection by e-beam–assisted Pt deposition from a metal-organic precursor gas on the top and front side faces, followed by ion beam–assisted Pt deposition on the top face. Lamellae were extracted such that the depth of the final thinned lamella was 50 to 100 nm from the side surface of the cantilever; thinning was performed down to a final polish at 2 kV, 50 pA. The isolated point features in the image, which are known to result from FIB damage (*67*)—from the original microcantilever fabrication here—were unavoidable as the liftout was purposely taken close to the cantilever surface to best reflect the dislocation structure resulting from the stress and strain state mapped on the cantilever side surface. TEM was performed on a JEM-2200FS (JEOL, Japan) operated at 200 kV in bright-field scanning (BF-STEM) mode with a convergence semiangle of 10.8° and a 600-mm camera length. Nonlinear adjustment (gamma correction) was applied to the images.

### Extraction of total dislocation density

The total dislocation density was measured from BF-STEM images acquired close to the [001]_W_ zone axis (see inset fig. S11A). On the basis of groundwork by Mills and co-workers (*68*) on diffraction contrast STEM and recent studies (*69*, *70*), this low index zone axis condition is effective to image the complete dislocation density in a single image, considering that the STEM image is hence constituted of the average contrast of all reflectors contained within the BF detector disc. Furthermore, imaging along the [001]_W_ axis has the additional benefit that the crack region is viewed with the same perspective as the strain mapping, and, hence, no distortion correction is required.

A line intercept method (*71*) was applied within subsets of equal size to the HR-EBSD scanning performed previously (75-nm square array, equating to 21 by 21 px^2^ subsets of the BF-STEM image; fig. S11). Intercept counting was semiautomated; following the manual tracing of dislocations, intersection of the traces with six straight lines within each subset was determined using a program written in MATLAB. Last, the total dislocation density ρ_tot_ is expressed as

$$\rho_{\text{tot}}=\frac{2N}{\mathrm{Lt}}$$ (1)

where the *N* is the number of intercepts between the dislocation array and the measurement lines of total length *L* per subset and *t* is the thickness of the TEM lamella. This assumes foil-penetrating dislocations. In fig. S11, a relative independence of the distribution of dislocation densities on the orientation of intersection lines is shown for two simple choices (vertical/horizontal and crossed 45°). The spatial variation in sample thickness across the height of the lamella was accounted for in the calculation of the dislocation density, assuming a linear thickness variation from the top (thinnest, 76 nm) to the bottom (thickest, 259 nm) of the lamella, measured using the STEM probe contamination method; no substantial lateral variation in the lamella thickness (standard error mean, 4 nm) was measured. This dislocation intercept method fails in proximity of the notch tip where the high dislocation density prohibits identification of individual dislocations—a well-known and unresolved limitation—hence, the measured dislocation density in this region should be considered a lower bound to the actual value.

### Atomic force microscopy

AFM was performed on a NTEGRA Spectra (NT-MDT, Russia) in semicontact (tapping) mode using standard Si tips (RTESPA-300, Bruker, USA).

## Supplementary Materials

### This PDF file includes:

Supplementary Text Figs. S1 to S12 Table S1 and S2 References
