# Supplementary material for: Mapping pure plastic strains against locally applied stress: Revealing toughening plasticity
Source: Sci Adv. 2022 Jul 27;8(30):eabo5735. doi: 10.1126/sciadv.abo5735 (PMC9328672; doi:10.1126/sciadv.abo5735)
Supplement: Supplementary file 1 — Supplementary Text Figs. S1 to S12 Table S1 and S2 References [file sciadv.abo5735_sm.pdf]

Science Advances
AAAS

Supplementary Materials for

Mapping pure plastic strains against locally applied stress: Revealing toughening plasticity

Thomas E. J. Edwards *et al.*

Corresponding author: Thomas E. J. Edwards, thomas.edwards@empa.ch

Sci. Adv. **8**, eabo5735 (2022)
DOI: 10.1126/sciadv.abo5735

This PDF file includes:

Supplementary Text
Figs. S1 to S12
Table S1 and S2
References

Supplementary Texts

1. Measuring strains: finite strain theory

Deformation gradients **F**, $\mathbf{F}_e$ and $\mathbf{F}_p$ may further be separated into strain (stretch & shear) and rotation components, as:

$$\mathbf{F} = \mathbf{RU} = \mathbf{VR} \qquad (2)$$

Where **R** is the rotation tensor, **U** is the right stretch tensor and **V** is the left stretch tensor. It can be shown(*73*) that $\mathbf{F}^{\mathrm{T}}\mathbf{F} = \mathbf{U}^2$ and $\mathbf{F}\mathbf{F}^{\mathrm{T}} = \mathbf{V}^2$, where T denotes the transpose. In the case of infinitesimal deformation, rotation and strain are approximated as summative, as is assumed for the HR-EBSD procedure here: $\mathbf{F}_{\mathrm{e}} = \mathbf{I} + \boldsymbol{\varepsilon}_{\mathrm{e}} + \boldsymbol{\omega}$, where **I** is the identity matrix, $\boldsymbol{\varepsilon}_e$ is the elastic strain tensor and $\boldsymbol{\omega}$ the lattice rotation. However in the case of plastic deformation, strains may be large, and as such finite strain theory must be applied to deformation measurements by DIC. The Seth-Hill family of deformation tensors describe a variety of methods to evaluate strain(*73*); the simplest, the Biot strain tensor approximates strain where strains and rotations are small:

$$\boldsymbol{\varepsilon}_{\mathrm{Biot}} = \mathbf{U} - \mathbf{I} \qquad (3)$$

More useful here, and used for plotting all DIC-based strain data in the present study, is the Green-Lagrangian strain tensor which is similar to simple engineering strain, but further accounts for the second order contributions to strain:

$$\boldsymbol{\varepsilon}_{\mathrm{Green-Lagrange}} = \frac{1}{2}(\mathbf{U}^2 - \mathbf{I}) \qquad (4)$$

Finally, for completeness, true strain may be robustly expressed for large strains using the Hencky strain tensor:

$$\boldsymbol{\varepsilon}_{\mathrm{Hencky}} = \ln(\mathbf{U}) \qquad (5)$$

2. Further information on strain mapping methods

In its simplest form, the measurement of elastic strain provides a single, volume averaged value for applied or residual elastic strain, e.g. hole drilling(*74*), X-ray(*75*) or neutron(*76*) diffraction measurement of lattice plane spacing. However, in Materials Science one is often interested in determining local spatial variations in elastic strains and small lattice rotations with high resolution, such as across semiconductor devices(*40*), and for other crystalline(*77*) and non-crystalline(*11*) material classes. Over the past few decades, mostly diffraction-based methods have answered this call: nano-beam synchrotron X-ray diffraction(*9*), various transmission electron microscopy (TEM) diffraction methods(*11,40*) and scanning electron microscopy-based high resolution electron backscatter diffraction (SEM HR-EBSD)(*10*), as well as atomic position imaging in the TEM(*78*). Accessible regions of interest range from hundreds of micrometres with resolution on the order of 100 nm(*79*), to mapping elastic strain and lattice rotation about individual dislocations(*80,81*).

For bulk values, total strain measurement may be achieved by a surface strain gauge or an extensometer, however mapping the local distribution of total strain may currently only be achieved by digital image correlation (DIC) methods – at a surface, by tracking relative movements in a reference pattern(*82*), and, rarely, by a similar approach in a volume(*83*). The recent emergence of SEM DIC(*13*) exploits the increased imaging magnifications of electrons over standard optics, but few studies achieve truly nanoscale strain mapping, nDIC, down to the tens of nanometre resolution(*14,15*) required to characterise slip lines(*37*) as this additionally depends on producing a suitably refined nanoscale surface pattern to follow. A known limitation of DIC techniques is that in principle, when applied with a single camera, a 2D projection of surface deformation is calculated; however, recent developments in DIC algorithms(*84*) demonstrate how this can be overcome in the case of discrete slip line-dominated plasticity, where deformation is constrained to a limited number of known crystal planes.

3. Further description of experimental results

Loading until the elastic limit

Elastic shear in the plane of the cantilever side surface, $\varepsilon_{e,12}$, additionally develops as two extended lobes either side of the notch tip with approximately equal and opposite values. Out-of-plane elastic strains ($\varepsilon_{e,13}$, $\varepsilon_{e,23}$ and $\varepsilon_{e,33}$) remain close to the noise threshold at the elastic limit.

Loading beyond the elastic limit

Normal elastic strain along the cantilever axis continues to increase at the notch tip, although $\varepsilon_{e,22}$ is higher (more than double, step 6) in the lobe to the left side than that on the right throughout the elastoplastic deformation regime. Contrastingly, in-plane elastic shear strain $\varepsilon_{e,12}$ increases more substantially along a ~45° path ahead of the notch tip on the right side, reaching ~1% at step 6, whilst the symmetric feature on the left is limited to ~0.5%. Although the other normal elastic strain components continue to display simple Poisson contraction, and changes to out-of-plane shear $\varepsilon_{e,23}$ are negligible, a substantial plume of heightened $\varepsilon_{e,13}$ elastic shear develops to the left of the notch; the reason for this is currently unclear.

The slip line operates at an in-plane elastic shear strain $\varepsilon_{e,12}$ upwards of 0.5% and the axial total strain $\varepsilon_{tot,22}$ generated therein continuously increases as the cantilever is further bent. In addition, a zone of more diffusely distributed total strain, where the strain levels far exceed those achievable by elasticity alone, additionally develops throughout a broad region stretching several microns wide and tall ahead of the notch.

Unloaded state after deformation

After unloading (step 7), all elastic strain components reduce significantly, by approximately 50%, and residual stress is symmetrically distributed about the notch tip; only the out-of-plane

$\varepsilon_{e,13}$ shear foregoes this trend, retaining the aforementioned heightened positive shear on the left side of the notch only.

4. Dislocation structures observed by transmission electron imaging

As might be expected, a concentration of linear crystal defects, dislocations, is observed below the notch tip, arranged in a complex network of interlocked tangles. Further, ladder-appearance features of dislocation pile-up extend outwards from the notch tip directed ~45° and ~60° to the horizontal, see skeleton-free TEM image in Fig. S11. The liftout geometry excludes the possibility that these ladders might be any mix of screw to edge type for the four $\{110\} < 1\bar{1}1 >$ slip systems in Fig. 3, whose planes and parallel to the viewing direction and intersect the side surface of the microcantilevers at 45° to the horizontal.

5. Discrepancy in dislocation density values measured by GND and TEM

The measured ρ_{GND} is found to have values approximately double those measured for ρ_{tot}; for a comparison on the same colour scale, see Fig. S7. One must note that the HR-EBSD calculation of GND content is sensitive to all dislocation types, including those mostly invisible to the TEM due to edge-on imaging here. However, on the other hand, the GND maps only measure a dislocation content required to fulfil the stepwise lattice rotations, with a 75 nm step here; the undetected, statistically stored dislocations, SSDs, are additionally imaged in transmission. BF-STEM imaging here is useful as contrast is generated from electron loss through (low-angle) coherent scattering by dislocations, whilst (low-to-high angle) mass contrast incoherent scatter and crystal orientation contrast are both minimised as the cantilever is a pure single crystal; hence the most complete dislocation population possible is captured, without significant contribution from other contrast sources. This differs with commonly used weak beam methods for dislocation density analysis, which only capture a specific subset of the total dislocation density; an extinction correction factor is then used(*85*), which spuriously places assumptions as to the uniformity of the distribution of dislocation types, and hence multiple imaging conditions of a same region may be required.

It is known that there is a minimum ρ_{GND} that can be measured, i.e. a noise threshold; this is higher (5.9×10^{13} m^{-2}) with the applied surface Pt speckle pattern here than without for tungsten(*25*) (5.1×10^{13} m^{-2}). This may be attributed to the base-level noise in the measurement of $\boldsymbol{\omega}$ rotation components, leading to a non-zero Nye tensor even before loading, which worsens with further mapping due to accumulating surface contamination. Similarly, the TEM intercept method is limited at the upper end of the range, when the dislocation array is highly dense; it is difficult to capture closely spaced dislocations in the skeletonisation. This is seen in the vicinity of the notch tip, Fig. 4C, where the measured ρ_{tot} is maximised at around 2×10^{15} m^{-2}.

Despite these differential lower and upper bounds, ρ_{GND} is nevertheless higher than ρ_{tot} throughout much of the conventional notch-tip plastic zone. This may be because ρ_{tot} reduces upon TEM lamella fabrication, by dislocation migration to the surfaces. However, unlike the low lattice resistance of some metals such as copper which results in room temperature annealing in short timescales(86), the high Peierls stress of tungsten(*87*) is thought to resist this annealing process. Alternately, one may also consider that the cantilever bending geometry here inherently leads to elastically-mediated lattice rotation preceding any local dislocation activity; hence a phantom elastic component may additionally contribute to the measured Nye tensor, artificially further raising the inferred GND density, as previously seen(*25*). Nevertheless, upon unloading (Fig. S7) this background contribution should mostly disappear. Finally, one may note in Fig. S7 that for the low dislocation density, elastically relaxed region of the cantilever root, far to the left of the notch, the logical ranking of $\rho_{tot} > \rho_{GND}$ is adhered to, despite this being at the maximum accumulation of surface contamination from repeated scanning. This observation suggests that the storage of dislocations itself is a major contributor, causing the Kikuchi pattern quality to diminish, and in turn the noise-induced fictitious GND density to increase. This is a universal concern for the application of HR-EBSD. However, it does not contradict key observations here, such as the symmetry of ρ_{GND} about the notch.

<u>6. Note on the apparent asymmetry of the notches</u>

One should note that the apparent asymmetry of the notch shape in elastic and total strain maps, Fig. 2 – 5, is simply a result of the 70° sample tilt. Imaging parallel to the crack, Fig. S1B, demonstrates this.

<u>7. Atomic force microscopy measurement of slip step</u>

Atomic force microscopy profiling, Fig. S5, of a repeat cantilever to that in Fig. 2 to avoid damage before liftout for transmission imaging similarly indicates a single surface slip step, emanating at ~45° to the south-east of the notch tip, with a decreasing height from the notch outwards; this matches well the corresponding DIC total strain map, Fig. S5C. At a reference distance of 0.6 μm from the notch tip along the slip line, the step height is 7.5 nm. Along this plane, two $\{110\} < 1\bar{1}1 >$ slip systems may be active (systems 1 & 2 in Fig. 3). The orientation of the slip step indicates a majority of the total slip here is carried by slip system 1 – perfectly equal amounts of slip in both in-plane $< 1\bar{1}1 >$ directions would yield a null surface slip step.

8. Extraction of the pure plastic component of deformation, $\mathbf{F}_p$

In theory, an obvious numerical approach to extracting the pure plastic deformation gradient tensor from the DIC and HR-EBSD data is to inverse the elastorotational gradient $\mathbf{F}_e$, and use this to left-multiply the total deformation gradient:

$$\mathbf{F} = \mathbf{F}_e\mathbf{F}_p \qquad (6a)$$

$$\mathbf{F}_p = \mathbf{F}_e^{-1}\mathbf{F} \qquad (6b)$$

In fact, it is expected that in most cases for engineering materials, the plastic strains that must be sustained for these to be considered 'damage tolerant' are sufficiently large that the contribution of elastic strains to the total are negligible(*88*); however, the lattice rotations are not.

In practice, one must consider the configurations in which the data is collected. For example, if one executes the digital image correlation procedure in the forward sense, that is the image of the deformed condition relative to that of the initial undeformed case (i.e. calculation of **F**), the output may be considered on either the regular grid of the initial image, or on the irregular, deformed grid, as illustrated in Fig. A.1 of (*8*). In Fig. 2 of the present study, the total strain data from **F** is plotted on the deformed grid, for example. For the extraction of $\mathbf{F}_p$, it is convenient if both the DIC and HR-EBSD data are plotted on a regular grid, with ideally the same pitch, to facilitate overlay.

From Fig. 1, it is apparent that the configuration common to both the total and elastorotational deformation is B, the final configuration. Therefore, this was chosen as the configuration on which to perform the calculation of $\mathbf{F}_p$, noting that $\mathbf{F}_p$ would therefore be plotted on a configuration corresponding to neither of its 'natural' states – i.e. neither B_0 nor B_p. In theory, it is possible to return the regular grid of $\mathbf{F}_p$ data to the B_0 or B_p configurations through a reverse transformation of the gridpoint positions using $\mathbf{F}^{-1}$ or $\mathbf{F}_e^{-1}$, respectively. This was not considered necessary in the context of the present study.

In order to generate a regular grid of datapoints in configuration B whilst avoiding introducing data processing artefacts and compounding error (i.e. limiting unnecessary data interpolation) the reverse digital image correlation between image pairs (initial and step X) was performed. Hence $\mathbf{F}^{-1}$ was instead calculated, based on a regular grid of correlation subsets in the deformed configuration at loading step X. Similarly HR-EBSD scans produced, by default, a regular grid of datapoints in the deformed configuration. With this in mind, it is more judicious to consider an alternative expression of Eq. 6a, as used in the present study for the extraction of $\mathbf{F}_p$:

$$\mathbf{F}_p = (\mathbf{F}^{-1}\mathbf{F}_e)^{-1} \qquad (7)$$

as this minimises the number of inversion operations required compared to Eq. 6b, which would require the inversion of both measured $\mathbf{F}^{-1}$ and $\mathbf{F}_e$ independently.

$\mathbf{F}_e$ was compiled according to:

$$\mathbf{F}_e = \mathbf{I} + \boldsymbol{\varepsilon}_e + \boldsymbol{\omega} \qquad (8)$$

where **I** is the identity matrix, $\boldsymbol{\varepsilon}_e$ is the elastic strain tensor and $\boldsymbol{\omega}$ the lattice rotation, as in Fig. 2. $\mathbf{F}^{-1}$ was compiled as:

$$F^{-1}{}_{ij} = \delta_{ij} + \frac{\partial u'_j}{\partial x_j} \qquad (9)$$

where δ_{ij} is the Kronecker delta and $\mathbf{u'}$ is the displacement field that relates the position of elements in the initial state, relative to their position in the deformed state. $d\mathbf{x}$ is a line element in the deformed state, and $d\mathbf{X}$ (Fig. 5) equivalently in the initial state, i.e. $d\mathbf{X} = \mathbf{F}^{-1}d\mathbf{x}$, as in (*8*).

As the SEM-DIC strain mapping method only allows the in-plane strain components to be calculated, $\mathbf{F}_p$ was similarly restricted: therefore only the four 1,2 indices components of $\mathbf{F}_e$ were used for the calculation of $\mathbf{F}_p$.

The magnifications for SEM imaging of the Pt surface speckle pattern were chosen such that one of the common binary subset sizes would be 75 nm wide, or simple fractions or multiples thereof, e.g. for the smaller cantilever size, 16 px = 75 nm. Where necessary regridding of the $\mathbf{F}^{-1}$ field could then be performed using bi-cubic interpolation based on standard Matlab functions.

9. An annealed slip band

The concept of an annealed slip band, along which mobile dislocations move rapidly before exiting the sample at a surface is supported by the absence of reverse plasticity along this line upon unloading of the cantilever and the low total dislocation density in this location. Unlike the distributed dislocation array captured by GND mapping, most dense at step 6, Fig. 4, but of which some nevertheless remains upon unloading, the very large operative dislocation population responsible for the intense right-hand slip line has been lost – reverse plasticity there would require significant further nucleation of dislocations, for which stress accumulation upon unloading is apparently insufficient to motivate it. Indeed, one should consider that the, at least, 700-strong population of mobile dislocations identified in Fig. S10 to generate the southeastern plasticity could not simply be stored within the cantilever, as this would equate to an average dislocation density of 2×10^{15} m^{-2} along the entire slip band, which is clearly not measured by TEM, Fig. S11.

10. Analysis of results based on finite element crystal plasticity theory, and other models

Within finite element theory, that is the step-wise partitioning of space, as applied to crystals(*73*) (e.g. CPFEM), dislocation populations may be subdivided into geometrically necessary dislocations (GNDs) and statistically stored dislocations (SSDs, density ρ_{SSD}). Whilst the former fulfil the geometrical incompatibilities in the plastic deformation of adjacent cells, i.e. Curl $\mathbf{F}^p \neq 0$, measured as rotation of the crystal lattice in the step-wise HR-EBSD mapping, the latter are arranged in such a fashion that no net lattice rotation is generated between neigh-

bouring cells by this population. This classification of dislocations is not fundamental; however, it does serve in the understanding of the observations here of GND and total dislocation contents, and how this may relate to plasticity. The fact that after unloading the 45° band of intense plasticity and high ρ_{GND} on the right side of the notch tip displays no salient features on the ρ_{tot} map, whereas the low-plasticity, high ρ_{GND} 45° band on the left presents a raised total dislocation density, indicates a disparate distribution of SSDs. Hence ρ_{SSD} in the left-hand band must be higher than on the right side of the notch tip. Accurate quantification of this ρ_{SSD} difference is prohibited for reasons in Supplementary Material 5. It is interesting to note that within this finite element formulation, the population of mobile dislocations $\rho_{\mathrm{m}}^{\alpha}$ is proportional to the density of immobile, statistically stored, dislocations $\rho_{\mathrm{SSD}}^{\alpha}$ for a given slip system α (via a slip system-dependent interaction parameter) – the contribution of GND content to $\rho_{\mathrm{m}}^{\alpha}$ remains spatially symmetrical about the notch throughout testing. At the same time, the net shear rate $\dot{\gamma}^{\alpha} = \rho_{\mathrm{m}}^{\alpha} b v^{\alpha}$ and therefore depends on the dislocation velocity v^{α} which for the present discussion varies of significance as $v^{\alpha} \propto \sinh\left[A\left(\tau^{\alpha} - B\mu b\sqrt{\rho_{\mathrm{P}}^{\alpha} + \rho_{\mathrm{m}}^{\alpha}}\right)\right]$, hence:

$$\dot{\gamma}^{\alpha} \propto \rho_{\mathrm{m}}^{\alpha}\, b \sinh\left[A\left(\tau^{\alpha} - B\mu b\sqrt{\rho_{\mathrm{P}}^{\alpha} + \rho_{\mathrm{m}}^{\alpha}}\right)\right] \qquad (10)$$

where for positive applied shear stress τ^{α} on the slip plane, μ is the shear modulus, b the Burgers vector of slip, $\rho_{\mathrm{P}}^{\alpha}$ is the density of dislocation components parallel to α of all slip systems, which will hence induce a passing resistance to the movement of the mobile α dislocations that constitute $\rho_{\mathrm{m}}^{\alpha}$, and A and B are positive constants for fixed temperature and slip system type(*89*). Hence the effect of $\rho_{\mathrm{m}}^{\alpha}$ on the strain rate is not simply determined as v^{α} decreases with increasing $\rho_{\mathrm{m}}^{\alpha}$.

The present single crystal example demonstrates a certain non-physicality of this finite formulation: although a low, fast moving mobile dislocation population is experimentally found to be beneficial to plasticity, this low $\rho_{\mathrm{m}}^{\alpha}$ serves to repress the programmed multiplication processes in the CPFE formulation, e.g. dipole formation(*89*), whilst no dislocation loss at free surfaces or other sinks is usually envisaged either. The experimental result here would require a highly active population of dislocation sources along the dominant slip line in the CPFEM context – in reality, dislocations are likely mostly generated in the high stressed proximity of the notch tip. Comparison relative to existing CPFEM studies(*34,42*) of microcantilever bending using non-local formulations that allow GNDs and hence a length-scale to be introduced yields discrepancies concerning the role of GNDs. The storage of GNDs in such simulations(*34,42*) has been interpreted to impose an additional resistance to the motion of the mobile dislocations, resulting in small-scale strengthening effects. However, the present GND density symmetry and plastic asymmetry would suggest this description is incomplete. In such CPFEM cases, the nucleation rate and spatial distribution of sources, as well as the possible starvation of mobile dislocations, are not considered.

Discrete dislocation models, which do account for dislocation nucleation and loss of the individual dislocations, have similarly been applied to microcantilever bending(*28*); equivalently to GNDs, nucleation source density was found to strongly control small scale strengthening effects. However, the simulated Frank-Read sources operated when a threshold stress was locally exceeded for a prescribed time; hence a variable rate of activation of the source has not been robustly considered.

11. Discussion points concerning loading- or strain-rate

It was necessary, here, to pause in situ loading (otherwise at 0.05 MPa $m^{1/2}$ s^{-1} for the 5 μm height cantilever, 0.06 MPa $m^{1/2}$ s^{-1} for the 3 μm one; these are relatively slow rates(*22*)) for 20 min at each strain mapping step for diffraction map and speckle image acquisition, under load: effectively a variable loading rate fracture test. In theory, for BCC tungsten at room temperature, some dislocation motion can occur during these holding periods; nevertheless, the coherency in J-integral against crack length when comparing with continuous loading (Fig. S1) indicates the pauses have little effect on the deformation mechanistics and cracking during the dynamic phases.

12. Discussion points concerning the toughness of tungsten

Current understanding of fracture processes across brittle to ductile transitions (BDT) find consensus in crack tip plasticity in crystals being a two event process(*90*). For effective toughening, it requires both efficient dislocation nucleation and rapid glide i.e. high dislocation mobility within the plastic zone(*44*). Previous seminal work on tungsten(*45*) found nucleation to be limiting only well below the BDT, by cryogenic testing after different amounts of cold work-induced dislocation source arrays were introduced. In the transition regime, and at higher temperatures, dislocation mobility controlled crack tip toughening processes.

13. Size effects study

Smaller notched cantilevers, 3 μm in height, were equivalently deformed, Fig. S9. The conditional fracture toughness was lower for these reduced dimensions, 5.1 MPa $m^{1/2}$, as was the maximal angle of rotation of the beam relative to the root before rapid crack growth. The magnitude of plastic strain achieved below the notch was similarly reduced compared with the larger cantilevers; however, the asymmetry of the distribution of strains and lattice rotations relative to the notch was maintained, albeit more subtly. Plasticity is observed to focus on the right side of the notch, whilst the normal stress along the cantilever axis is a maximum ahead of the notch on the cantilever root side. Finally, the region of heightened GND density forms a vertical band, following the crack propagation direction, and displays little lateral spread, unlike the notch plume in the 5 μm cantilever.

Although elastic stresses and strains are similarly distributed during loading of both notched cantilever sizes, the lateral distribution of geometrically necessary dislocations is considerably lesser in the smaller sample and achieves lower maximum densities, yet remains symmetrical about the notch: dislocation multiplication initiates at the notch tip and GNDs extend only as far as soft pile-up at the axial tension-compression boundary (see σ_{22} component) permits.

Supplementary Figures

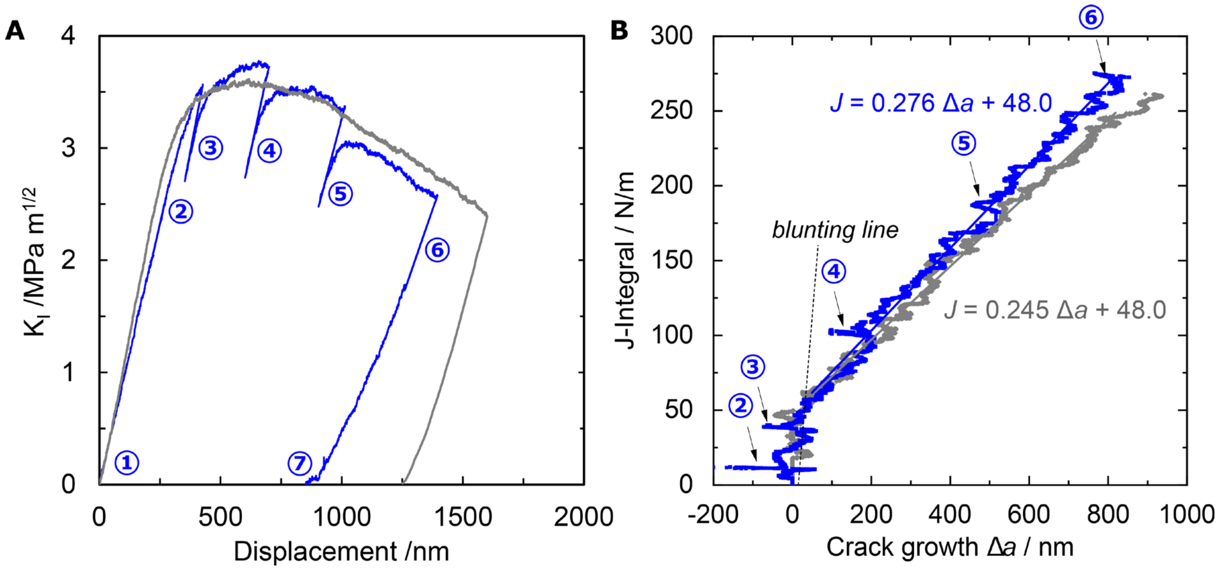

Fig. S1: Comparison of strain mapped and continuous deformation cantilevers. Comparison of current notched cantilever loading here (larger cantilever size, Fig. 2, blue) with previous data (grey) acquired without interruptions, showing good agreement in both (**A**) loading curves and (**B**) J-integral crack growth lines. In (**A**), the mode I stress intensity factor at the crack tip calculated based on linear elastic fracture mechanics (LEFM)(*22*) is plotted against displacement of the sphero-conical tip contact point. Note that although this LEFM assumption is only truly valid until the elastic limit (step 2), it serves as an effective method to compare crack tip stress states, normalising any differences in exact cantilever geometry between the two testpieces, such as in loading arm length. (**B**) confirms that no measurable crack growth occurs during the displacement holding periods for acquisition of EBSD maps and speckle pattern images. The conditional fracture toughness measured using the plasticity-considering J-integral method for the larger cantilever size here, 6.8 MPa $m^{1/2}$ at 0.05 MPa $m^{1/2}$ s^{-1}, lies comfortably within the 6.6 – 7.0 MPa $m^{1/2}$ range previously determined(*24*) for stress intensity factor rates of 0.03 – 0.1 MPa $m^{1/2}$ s^{-1}.

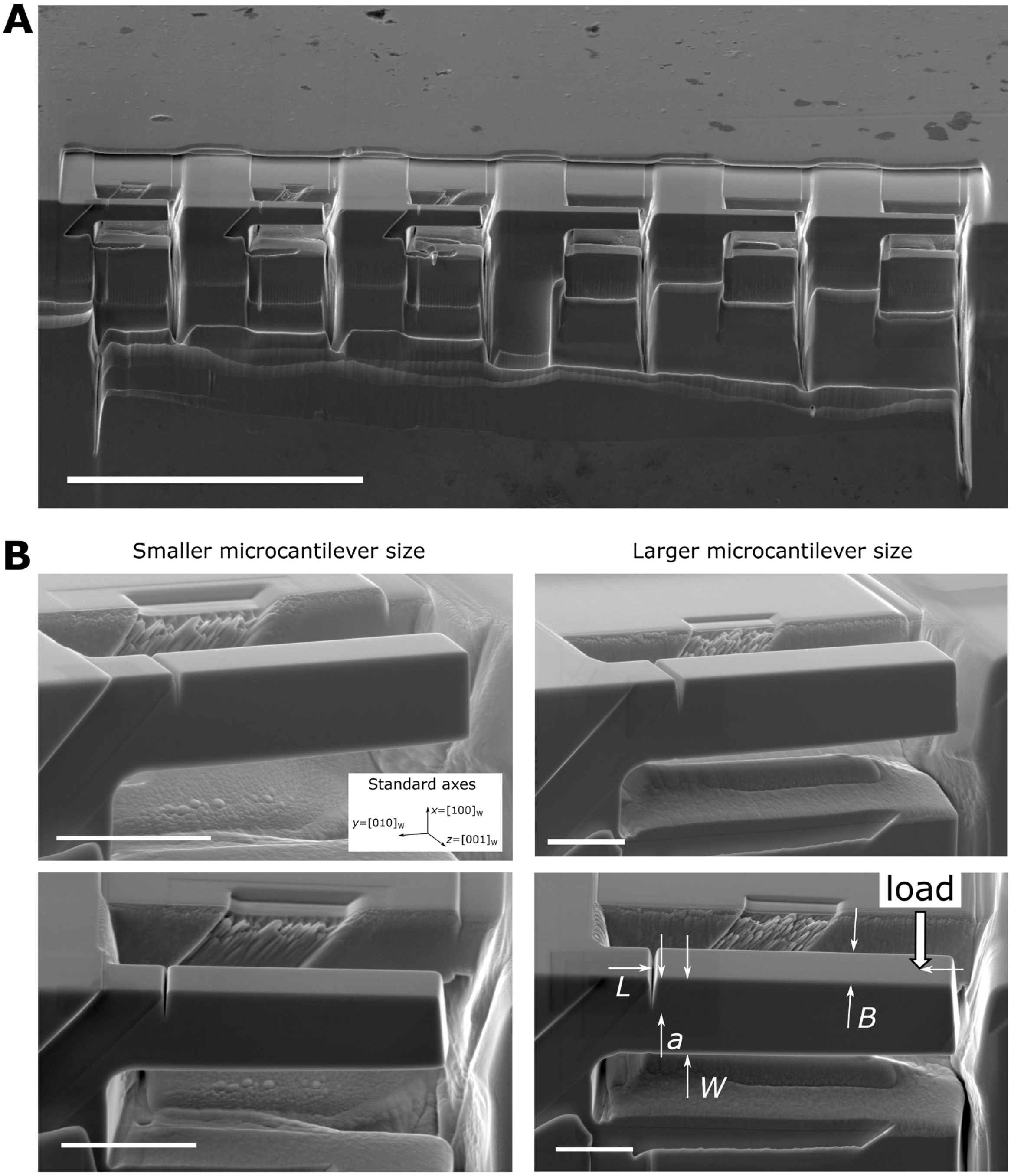

Fig. S2: Micromechanical testpiece geometries.
(**A**) Secondary electron SEM images of the FIB-machined arrays of pure W (100)-(010)-(001) rectangular cantilevers (smaller cantilever geometry) and (**B**) higher magnification images of both cantileved sizes. Dimensions *W*, *B*, *L* and *a* are reported in Table S1 for both cantilever sizes. The scale bar in (**A**) is 50 μm long; the scale bars in (**B**) are 5 μm long.

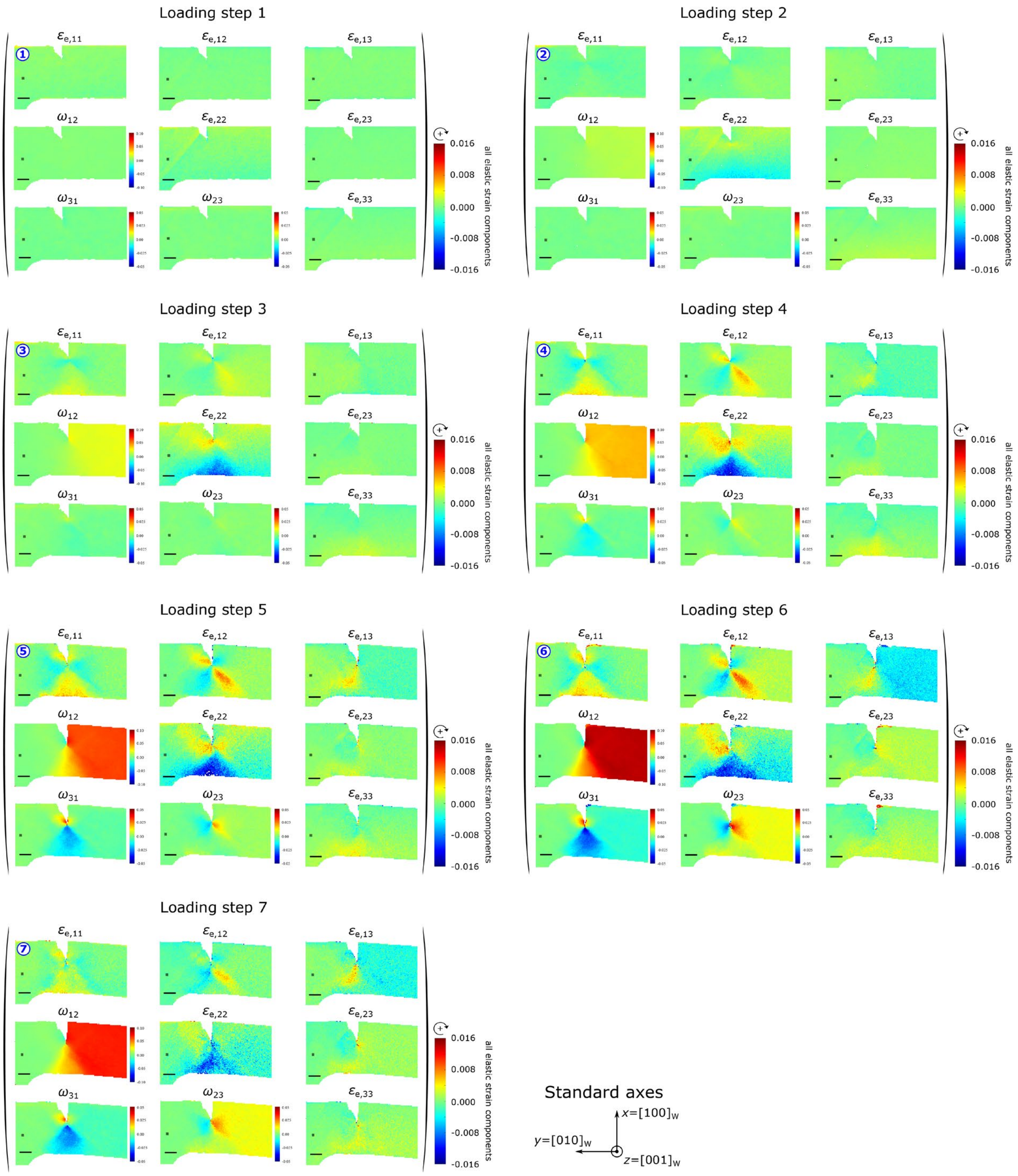

Fig. S3: Further elastic strain and lattice rotation mapping data for the larger cantilever geometry.

This considers elastic strain $\boldsymbol{\varepsilon}_e$ and lattice rotation $\boldsymbol{\omega}$ tensors. The scale bars are 1 µm long.

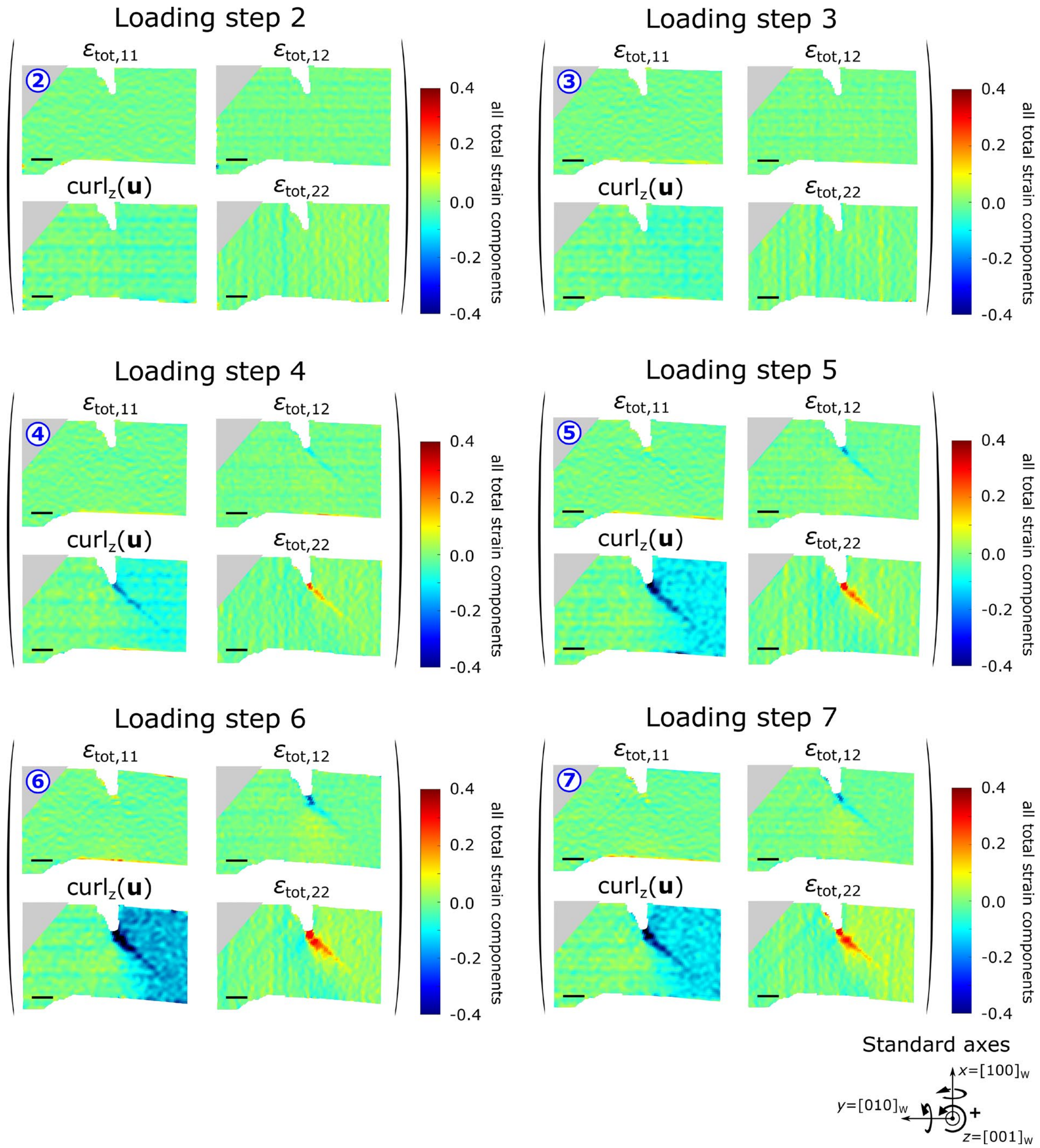

Fig. S4: Further total strain and rotational component mapping data for the larger cantilever geometry.

This considers total strain $\boldsymbol{\varepsilon}_{tot}$ tensor & rotational derivatives. The scale bars are 1 µm long.

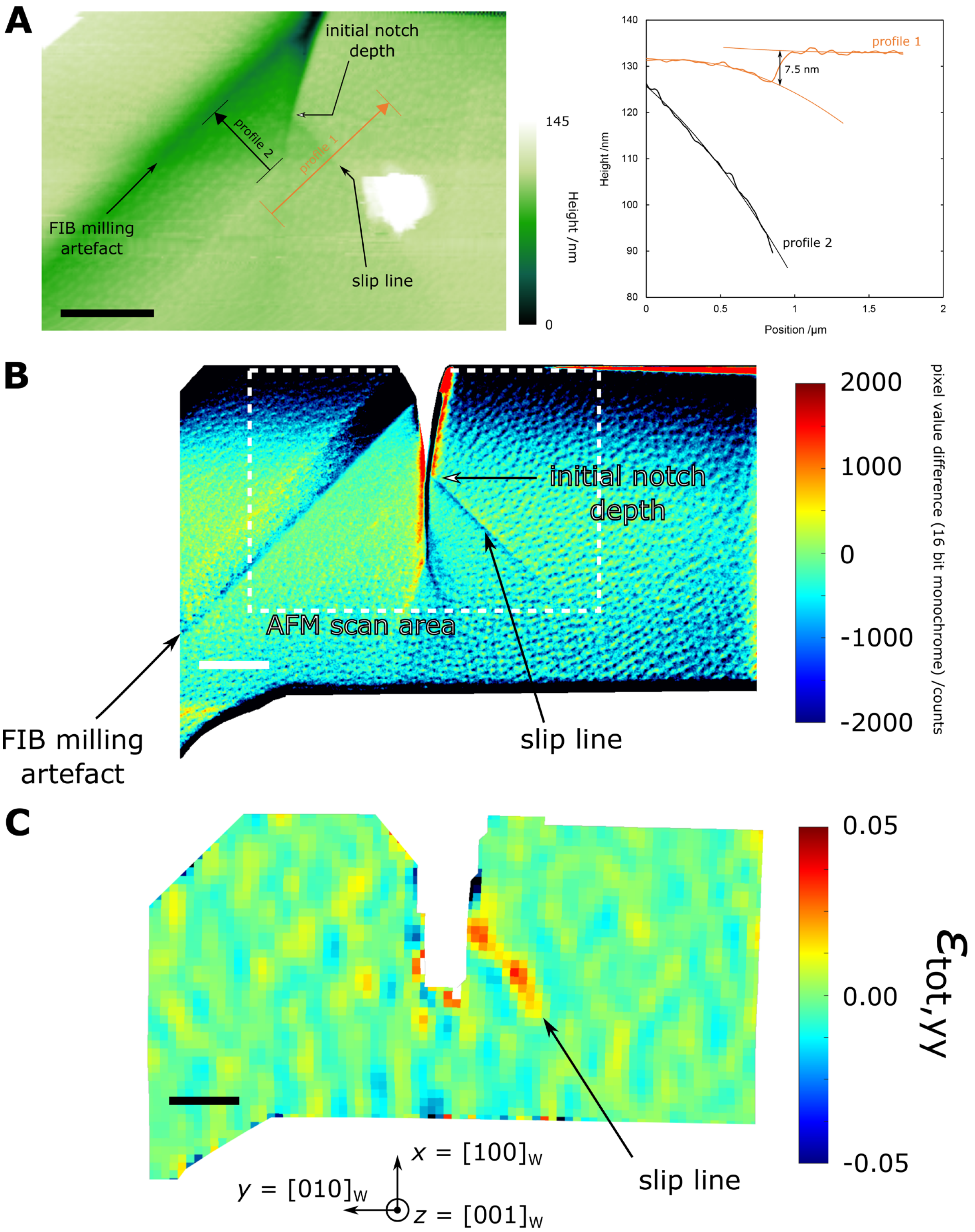

Fig. S5: Atomic force microscopy of the slip band ahead of a notch tip.
(**A**) Atomic force microscopy scan of the root-notch region of a large size cantilever, equivalent to that in Fig. 2; it was not possible to scan the exact same cantilever due to the eventual damage caused by AFM scanning, which should not compromise the possibility of obtaining the liftout sample for TEM imaging, Fig. 5. The scan step size was 20 nm. The Pt surface speckle pattern is visible across the entire scan area. Profiles (black and orange) averaged across a 20 px width are extracted on the right; the height of the step across the slip line (profile 1) was measured to be 7.5 nm. A second degree polynomial was fit to the profile segments as a guide to the eye; non-planarity, particularly profile 2, was due to the

unavoidable FIB milling artefact near the notch. The white patch mid-right is surface contamination. (**B**) For the larger size cantilever in (**A**), the difference between the pixel values of the initial (undeformed) and final (unloaded, after deformation) SEM images of the sample surface is plotted. The slip line extending at ~45° south-east from the initial notch-tip position is clear; no equivalent line is observed here, or by AFM (profile 2), extending at ~45° south-west of the notch tip. A 5 × 5 px^2 Gaussian smoothing kernel was applied to the differential image. The dashed white rectangle in (**B**) delimits the AFM scanned area in (**A**). (**C**) Total normal strain map along the cantilever axis for the same cantilever as in (**A**,**B**); the final (eighth), unloaded step is presented here. The slip line heading southeast from the notch tip is again visible. The map is lower resolution than those in Fig. 2 due to increased imaging noise: the DIC subset size was 169 × 169 nm^2 (50% overlap). The scale bars are 1 μm long.

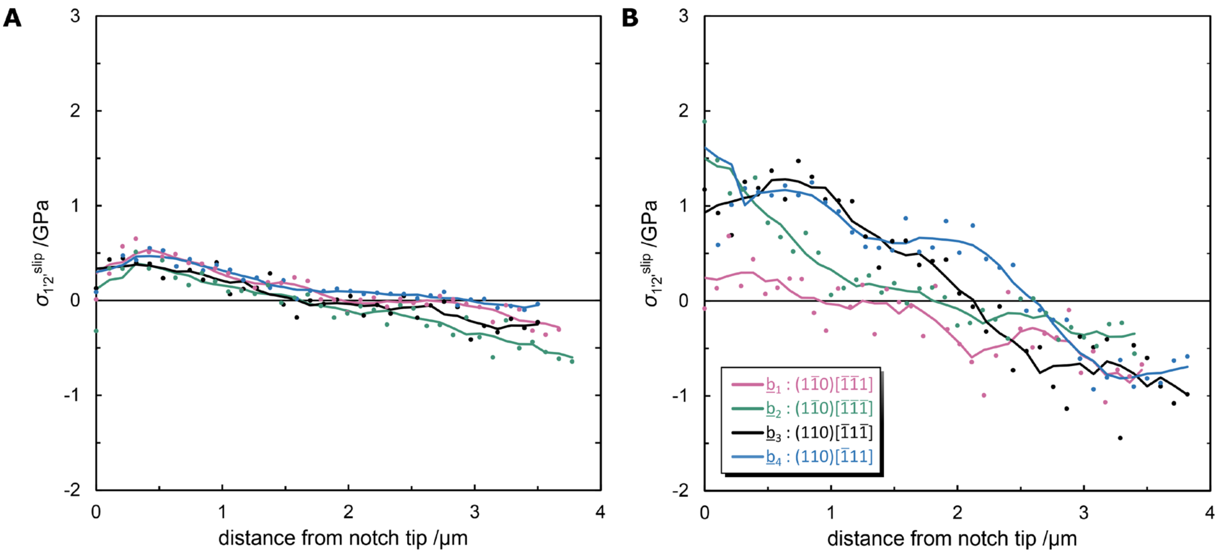

Fig. S6: Raw data and 5 neighbouring point centred averaging line for the shear stress resolved onto the four slip systems in Fig. 3.
This is given at (**A**) the elastic limit (loading step 2) and (**B**) within the plastic regime (step 5). The error in $\sigma_{1'2'}^{slip}$ per datapoint is 0.16 GPa, by propagation of uncertainty upon axes rotation, using the errors of all stress components in the measurement axes, see Methods. All scale bars are 1 µm long.

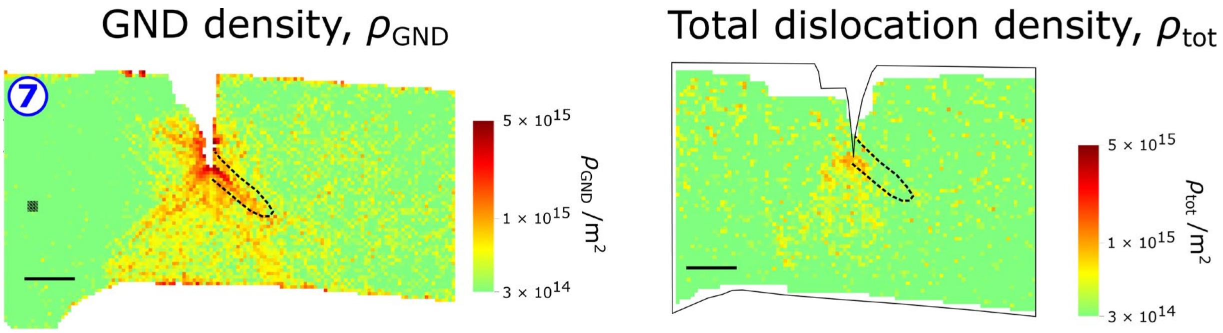

Fig. S7: Comparison of GND (unloaded, step 7) and total dislocation density maps on the same colour scale.

Considerably lower total dislocation densities are measured below the notch than GNDs, indicating either an extensive loss of dislocations upon thin film extraction, or an overestimation of GNDs in the energy-minimisation of the Nye tensor solution method(*30,31*). The scale bars are 1 µm long.

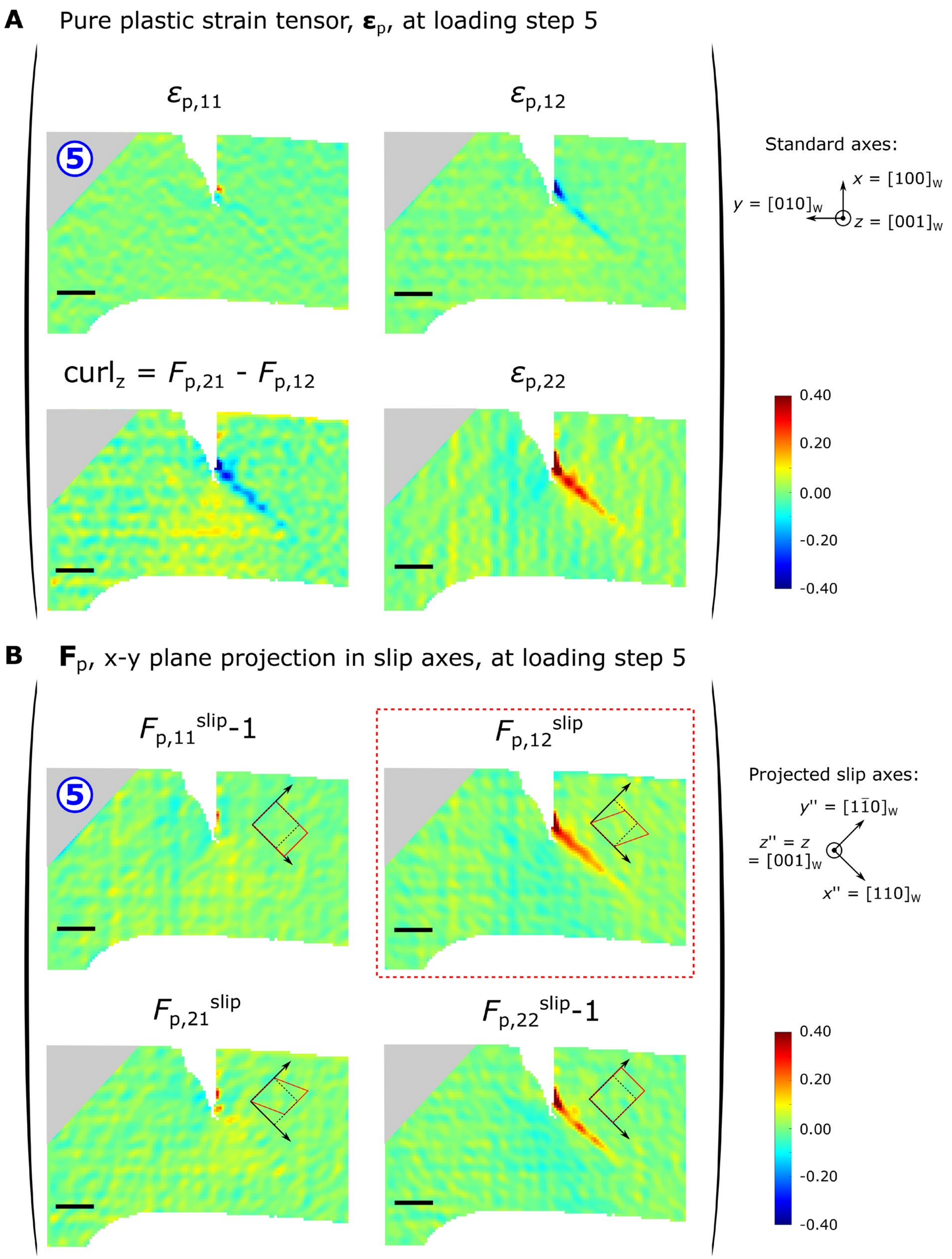

Fig. S8: Pure plastic component of deformation, $\mathbf{F}_p$, and pure plastic strain, ε_p, at loading step 5.

(A) The Green-Lagrangian $\boldsymbol{\varepsilon}_p$ components and (**B**) the $\mathbf{F}_p$ (dimensionless) ones are plotted on the deformed shape, owing to a facilitation of the analysis procedure that this entailed: see Supplementary Material 8. Insets indicate the type and positive sense of deformation per $\mathbf{F}_p$ component (dotted black square deforms to red rectangle/rhombus). The $F_{p,22}^{slip}$ expansion normal to the slip direction on the southeast line results from the 70° imaging angle, Fig. 1,

and confirms the direction of the slip step (see Fig. S5), whilst also suggesting opportunities for future improvements of the correlative strain mapping setup, such as stage tilt back-and-forth at each loading step to image the speckle pattern normal to the cantilever surface of interest. The pitch of the square grid for $\mathbf{F}_p$ maps is 75 nm. All scale bars are 1 μm long.

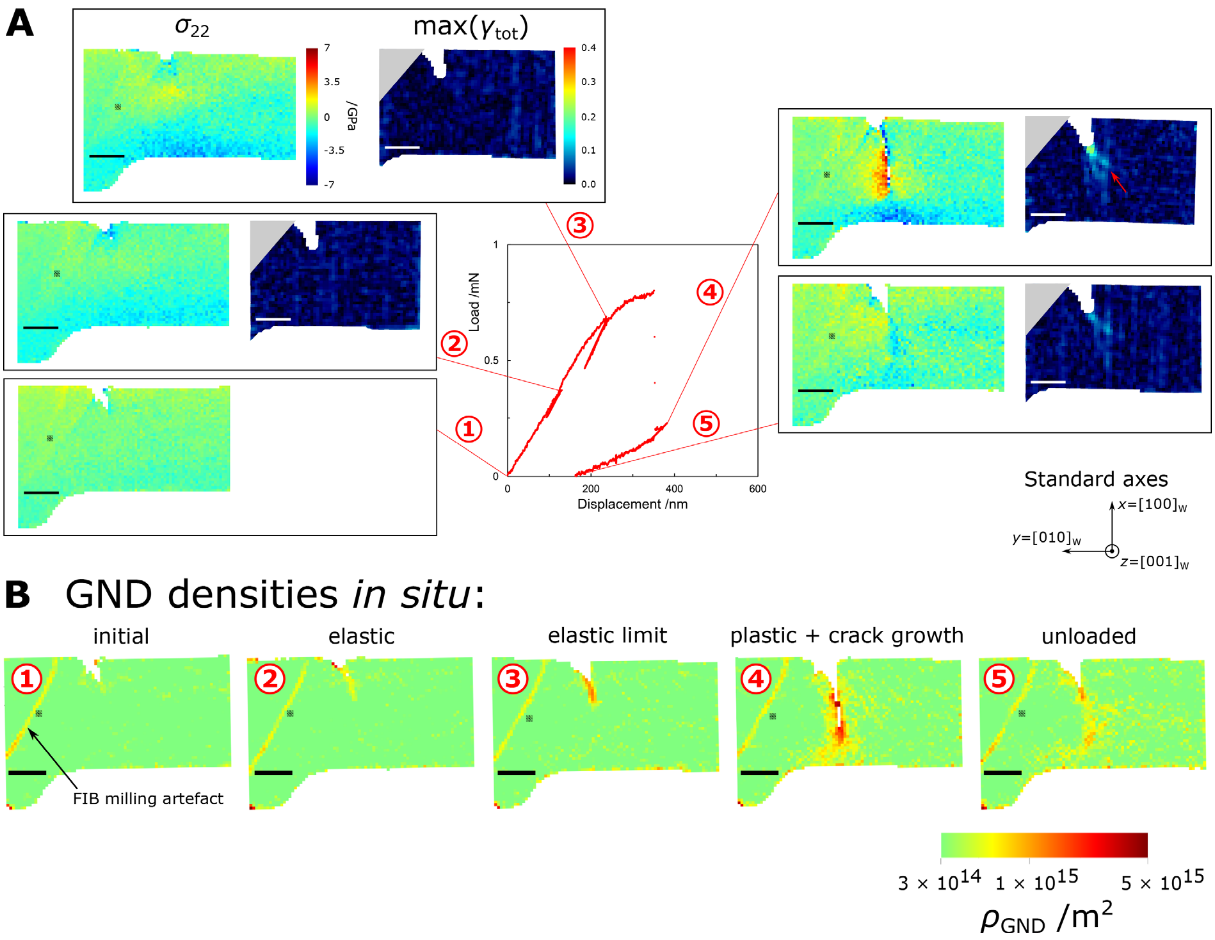

Fig. S9: Compilation of stress and total strain data for a (smaller) 3 µm height microcantilever.

(**A**) Equivalently to Fig. 2, the axial normal stress along the cantilever length and the maximum total shear strain is measured at several steps during loading. Between loading steps 3 and 4, rapid crack growth occurs; relatively little plastic deformation is observed to accumulate prior (strain scale retained from Fig. 2 for comparison), although asymmetry of plasticity is again observed at this size scale, being more intense southeast of the notch, arrowed in red. (**B**) The accumulation of geometrically necessary dislocations ahead of the notch is similarly measured to be more conservative than the larger cantilever, both in lateral spread and intensity. This coheres well with the lower measured toughness for the 3 µm cantilevers, resulting from lesser crack tip plasticity. The spatial resolution for HR-EBSD-based data is 75 nm; total deformation was presently calculated with 24 × 24 px^2 subsets with 25% overlap, yielding an 84.4 nm subset spacing. Errors of measurement for all quantities are again given in full in Methods. All scale bars are 1 µm long.

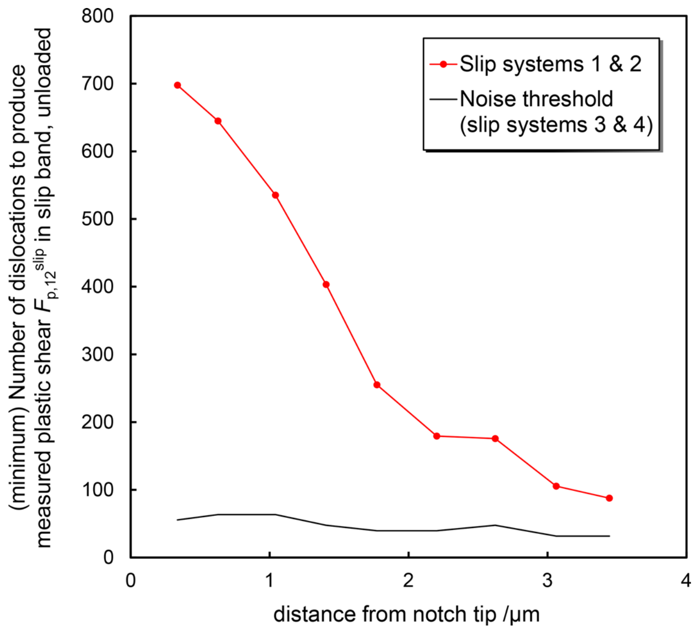

Fig. S10: Extraction of the number of dislocations passing from the plastic shear $F_{p,12}^{slip}$ at loading step 7 (unloaded).

The displacement parallel to and generated by the slip band emanating southeast of the notch in Fig. 2 was determined at ~400nm spaced positions along the slip band by integrating $F_{p,12}^{slip}$ over the band width along lines perpendicular to the slip band, which itself is aligned with the $(1\bar{1}0)$ plane trace (corrected for lattice rotation to the right of the notch upon cantilever bending, as in Fig. 3). The displacement (projected onto the $(001)_W$ side surface of the cantilever) was converted into a number of $\{110\} < 1\bar{1}1 >$ dislocations passing (slip systems 1 & 2 of Fig. 3) by dividing by the relevant Burgers vector b = 0.2741 nm and considering the $\sqrt{3/2}$ geometric correction for 2D strain mapping of the $(001)_W$ surface. The noise threshold for dislocation counting by this method was determined from the error in $F_{p,.12}$: calculated as in Fig. S12, for loading step 7 here: 0.18. The dislocation counting noise threshold varies with distance from the notch tip due to variations in the considered slip band width at each position along the slip band; it can be considered an upper bound to the number of dislocations that could have evolved along a southwest slip band (slip systems 3 & 4).

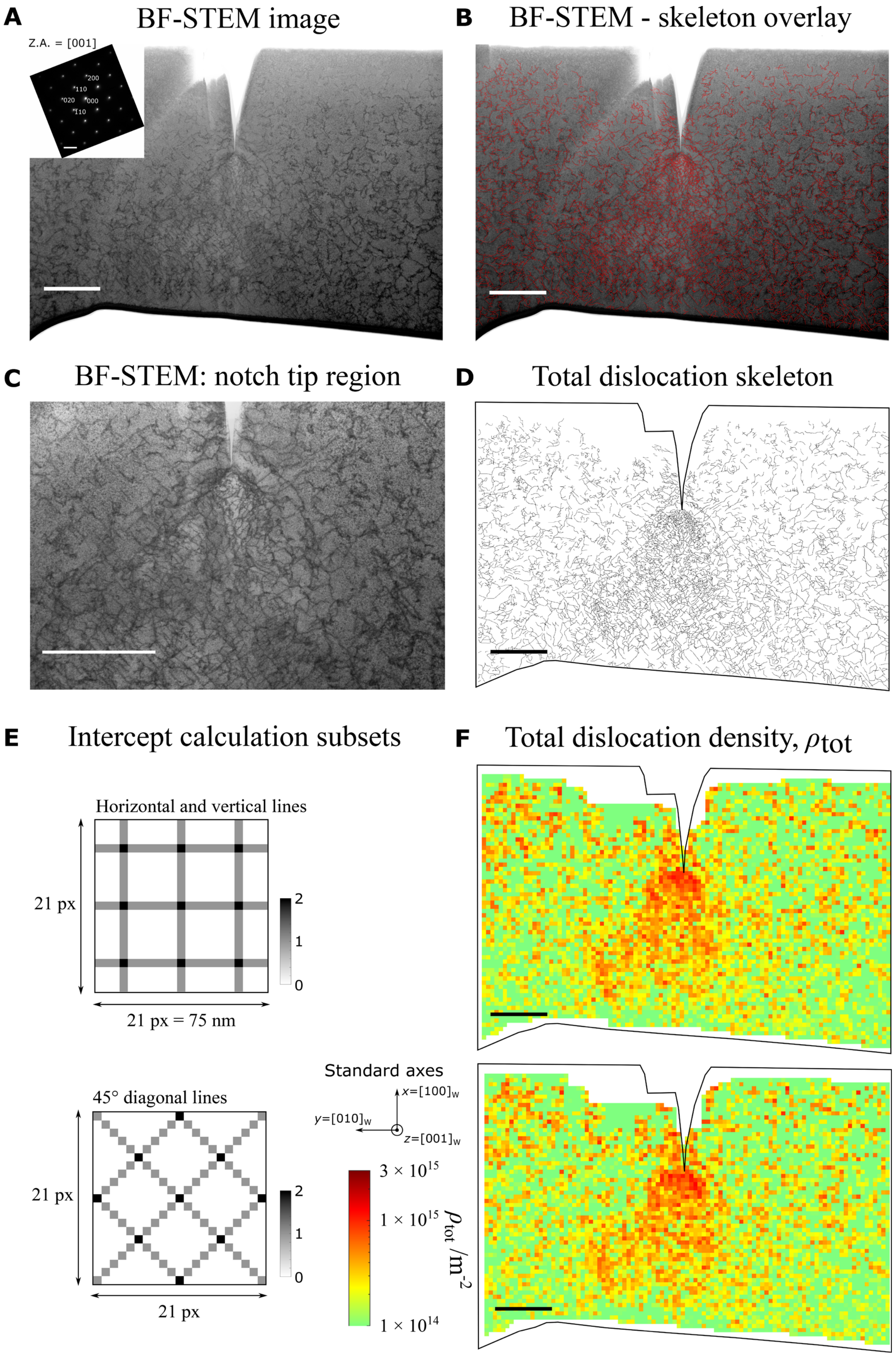

Fig. S11: Extraction of the total dislocation density from transmission electron imaging. (**A**) The thin film liftout obtained by FIB method from only 50-100 nm depth of the front,

strain mapped surface was imaged by bright field scanning TEM. This aimed to capture the most dislocations possible (in contrast to alternative weak-beam methods that selectively exclude certain dislocation orientations). A higher resolution image of the notch tip region is given in (**C**). Diffuse background contrast variations result from lifting the lamella close to the FIBbed surface, rather than unoptimised preparation for TEM. Following manual skeltonisation of the dislocation array (**B**,**D**), two masks for intercept measurement were created (**E**) with horizontal and vertical lines, or 45° diagonal lines. The mask size was 21×21 px^2 = 75×75 nm^2, i.e. equal to the HR-EBSD scan step. Intercepts between the masks and the dislocation skeleton were calculated using a Matlab code; from this the dislcoation density maps (**F**) were produced for both masks. The close similarity of the two ρ_{tot} maps demonstrates the robustness of the method relative to the choice of mask. The scale bars are 1 μm long; that for the diffraction pattern is 4 nm^{-1}.

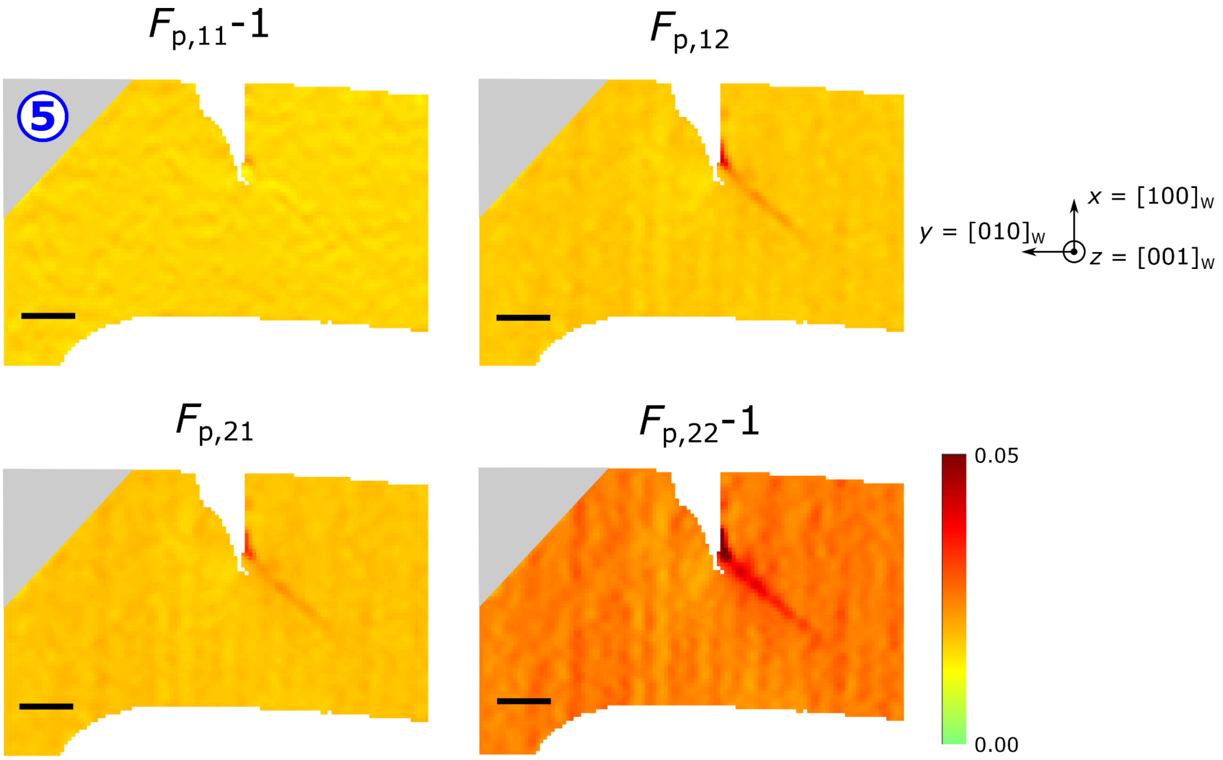

Fig. S12: Error values for the F_p components.
Based on the standard noises of measurement determined for HR-EBSD and DIC data, the propagation of uncertainty into the calculation of $\mathbf{F}_p$ was performed according to (*91*). The matrix inversion procedure results in higher absolute error along the line of extensive slip than in the remainder of the cantilever; the relative error is lower however along the slip line for $F_{p,12}$ and $F_{p,22}$-1. The scale bars are 1 µm long.

Table S1: Cantilever dimensions for the specific cantilevers in Fig. 2 and S9; see Fig. S1 for illustration.

Cantilever	Height, W / µm	Width, B / µm	Crack length, a / µm	Lever arm, L / µm
Larger, Fig. 2	5.1	4.8	2.1	16.5
Smaller, Suppl. Fig. 9	3.3	2.9	1.1	9.4

Table S2: Speckle pattern deposition parameters. Electron beam deposition parameters for the Pt speckle pattern suitable for combined DIC and HR-EBSD strain mapping on pure W. The bitmap image for pattern deposition is given in (*8*).

Beam voltage /kV	Beam current /nA	Dwell time / $ms.px^{-1}$	Number of passes	Duration /s	Pixels per speckle /px^2	Specified size of bitmap image /μm^2	Speckle particle diameter /nm
15	1.6	1.4	1	42	2 × 2	6 × 12	40 – 50

REFERENCES AND NOTES

1. E. O. Hall, The deformation and ageing of mild steel: III Discussion of results. *Proc. Phys. Soc. B* **64**, 747–753 (1951).

2. N. J. Petch, The cleavage strength of polycrystals. *J. Iron Steel Inst.* **174**, 25 (1953).

3. Y. Li, A. J. Bushby, D. J. Dunstan, The Hall–Petch effect as a manifestation of the general size effect. *Proc. R. Soc. A Math. Phys. Eng. Sci.* **472**, 20150890 (2016).

4. Z. C. Cordero, B. E. Knight, C. A. Schuh, Six decades of the Hall–Petch effect – a survey of grain-size strengthening studies on pure metals. *Int. Mater. Rev.* **61**, 495–512 (2016).

5. Y. Cui, G. Po, P. Srivastava, K. Jiang, V. Gupta, N. Ghoniem, The role of slow screw dislocations in controlling fast strain avalanche dynamics in body-centered cubic metals. *Int. J. Plast.* **124**, 117–132 (2020).

6. P. R. Howie, S. Korte, W. J. Clegg, Fracture modes in micropillar compression of brittle crystals. *J. Mater. Res.* **27**, 141–151 (2012).

7. R. J. Asaro, Crystal plasticity. *J. Appl. Mech.* **50**, 921–934 (1983).

8. F. Di Gioacchino, W. J. Clegg, Mapping deformation in small-scale testing. *Acta Mater.* **78**, 103–113 (2014).

9. G. E. Ice, J. D. Budai, J. W. L. Pang, The race to x-ray microbeam and nanobeam science. *Science* **334**, 1234 (2011).

10. A. J. Wilkinson, G. Meaden, D. J. Dingley, High resolution mapping of strains and rotations using electron backscatter diffraction. *Mater. Sci. Technol.* **22**, 1271–1278 (2006).

11. C. Gammer, C. Ophus, T. C. Pekin, J. Eckert, A. M. Minor, Local nanoscale strain mapping of a metallic glass during in situ testing. *Appl. Phys. Lett.* **112**, 171905 (2018).

12. V. B. Ozdol, C. Gammer, X. G. Jin, P. Ercius, C. Ophus, J. Ciston, A. M. Minor, Strain mapping at nanometer resolution using advanced nano-beam electron diffraction. *Appl. Phys. Lett.* **106**, 253107 (2015).

13. A. D. Kammers, S. Daly, Digital image correlation under scanning electron microscopy: Methodology and validation. *Exp. Mech.* **53**, 1743–1761 (2013).

14. A. Orozco-Caballero, D. Lunt, J. D. Robson, J. Quinta da Fonseca, How magnesium accommodates local deformation incompatibility: A high-resolution digital image correlation study. *Acta Mater.* **133**, 367–379 (2017).

15. T. E. J. Edwards, F. Di Gioacchino, W. J. Clegg, An experimental study of the polycrystalline plasticity of lamellar titanium aluminide. *Int. J. Plast.* **118**, 291–319 (2019).

16. A. A. Griffith, The phenomena of rupture and flow in solids. *Philos. Trans. R. Soc. Lond.* **221**, 163–198 (1921).

17. F. Roters, P. Eisenlohr, T. R. Bieler, D. Raabe, *Crystal Plasticity Finite Element Methods in Materials Science and Engineering* (Wiley-VCH, 2010), pp. xi, 197 p.

18. M. J. Buehler, H. Gao, Y. Huang, Atomistic and continuum studies of stress and strain fields near a rapidly propagating crack in a harmonic lattice. *Theor. Appl. Fract. Mech.* **41**, 21–42 (2004).

19. E. Plancher, thesis, ENSAM, Paris (2015).

20. J. Jiang, T. Zhang, F. P. E. Dunne, T. B. Britton, Deformation compatibility in a single crystalline Ni superalloy. *Proc. R. Soc. A* **472**, 20150690 (2016).

21. C. Zhao, D. Stewart, J. Jiang, F. P. E. Dunne, A comparative assessment of iron and cobalt-based hard-facing alloy deformation using HR-EBSD and HR-DIC. *Acta Mater.* **159**, 173–186 (2018).

22. J. Ast, J. J. Schwiedrzik, J. Wehrs, D. Frey, M. N. Polyakov, J. Michler, X. Maeder, The brittle-ductile transition of tungsten single crystals at the micro-scale. *Mater. Des.* **152**, 168–180 (2018).

23. T. Hirai, S. Panayotis, V. Barabash, C. Amzallag, F. Escourbiac, A. Durocher, M. Merola, J. Linke, T. Loewenhoff, G. Pintsuk, M. Wirtz, I. Uytdenhouwen, Use of tungsten material for the ITER divertor. *Nucl. Mater. Energy* **9**, 616–622 (2016).

24. J. Ast, M. Göken, K. Durst, Size-dependent fracture toughness of tungsten. *Acta Mater.* **138**, 198–211 (2017).

25. J. Ast, M. N. Polyakov, G. Mohanty, J. Michler, X. Maeder, Interplay of stresses, plasticity at crack tips and small sample dimensions revealed by in-situ microcantilever tests in tungsten. *Mater. Sci. Eng. A* **710**, 400–412 (2018).

26. J. Ast, G. Mohanty, Y. Guo, J. Michler, X. Maeder, In situ micromechanical testing of tungsten micro-cantilevers using HR-EBSD for the assessment of deformation evolution. *Mater. Des.* **117**, 265–266 (2017).

27. J. D. Carroll, W. Abuzaid, J. Lambros, H. Sehitoglu, High resolution digital image correlation measurements of strain accumulation in fatigue crack growth. *Int. J. Fatigue* **57**, 140–150 (2013).

28. E. Tarleton, D. S. Balint, J. Gong, A. J. Wilkinson, A discrete dislocation plasticity study of the micro-cantilever size effect. *Acta Mater.* **88**, 271–282 (2015).

29. J. Ast, B. Merle, K. Durst, M. Göken, Fracture toughness evaluation of NiAl single crystals by microcantilevers—A new continuous J-integral method. *J. Mater. Res.* **31**, 3786–3794 (2016).

30. T. B. Britton, A. J. Wilkinson, Stress fields and geometrically necessary dislocation density distributions near the head of a blocked slip band. *Acta Mater.* **60**, 5773–5782 (2012).

31. T. J. Ruggles, D. T. Fullwood, J. W. Kysar, Resolving geometrically necessary dislocation density onto individual dislocation types using EBSD-based continuum dislocation microscopy. *Int. J. Plast.* **76**, 231–243 (2016).

32. H.-J. Kaufmann, A. Luft, D. Schulze, Deformation mechanism and dislocation structure of high-purity molybdenum single crystals at low temperatures. *Cryst. Res. Technol.* **19**, 357–372 (1984).

33. J. R. Low, R. W. Guard, The dislocation structure of slip bands in iron. *Acta Metall.* **7**, 171–179 (1959).

34. A. Ma, F. Roters, D. Raabe, A dislocation density based constitutive model for crystal plasticity FEM including geometrically necessary dislocations. *Acta Mater.* **54**, 2169–2179 (2006).

35. F. Di Gioacchino, J. Quinta da Fonseca, An experimental study of the polycrystalline plasticity of austenitic stainless steel. *Int. J. Plast.* **74**, 92–109 (2015).

36. J. C. Stinville, N. Vanderesse, F. Bridier, P. Bocher, T. M. Pollock, High resolution mapping of strain localization near twin boundaries in a nickel-based superalloy. *Acta Mater.* **98**, 29–42 (2015).

37. T. E. J. Edwards, F. Di Gioacchino, H. P. Springbett, R. A. Oliver, W. J. Clegg, Stable speckle patterns for nano-scale strain mapping up to 700 °C. *Exp. Mech.* **57**, 1469–1482 (2017).

38. M. Legros, In situ mechanical TEM: Seeing and measuring under stress with electrons. *C. R. Phys.* **15**, 224–240 (2014).

39. M. N. Polyakov, R. L. Schoeppner, L. Pethö, T. E. J. Edwards, K. Thomas, B. Könnyű, X. Maeder, J. Michler, Direct co-deposition of mono-sized nanoparticles during sputtering. *Scr. Mater.* **186**, 387–391 (2020).

40. D. Cooper, T. Denneulin, N. Bernier, A. Béché, J.-L. Rouvière, Strain mapping of semiconductor specimens with nm-scale resolution in a transmission electron microscope. *Micron* **80**, 145–165 (2016).

41. D. Kuhlmann-Wilsdorf, Questions you always wanted (or should have wanted) to ask about workhardening. *Mater. Res. Innov.* **1**, 265–297 (1998).

42. E. Husser, S. Bargmann, The role of geometrically necessary dislocations in cantilever beam bending experiments of single crystals. *Materials* **10**, 289 (2017).

43. A. Giannattasio, Z. Yao, E. Tarleton, S. G. Roberts, Brittle–ductile transitions in polycrystalline tungsten. *Philos. Mag.* **90**, 3947–3959 (2010).

44. G. Michot, M. A. Loyola de Oliveira, G. Champier, A model of dislocation multiplication at a crack tip: Influence on the brittle to ductile transition. *Mater. Sci. Eng. A* **272**, 83–89 (1999).

45. P. Gumbsch, J. Riedle, A. Hartmaier, H. F. Fischmeister, Controlling factors for the brittle-to-ductile transition in tungsten single crystals. *Science* **282**, 1293–1295 (1998).

46. J. J. Möller, E. Bitzek, On the influence of crack front curvature on the fracture behavior of nanoscale cracks. *Eng. Fract. Mech.* **150**, 197–208 (2015).

47. E. Bitzek, *Atomistic Simulation of Dislocation Motion and Interaction with Crack Tips and Voids* (Shaker, 2006).

48. E. Bitzek, P. Gumbsch, Mechanisms of dislocation multiplication at crack tips. *Acta Mater.* **61**, 1394–1403 (2013).

49. J. Wang, A. H. M. Faisal, X. Li, Y. Hong, Q. Zhu, H. Bei, Z. Zhang, S. X. Mao, C. R. Weinberger, Discrete twinning dynamics and size-dependent dislocation-to twin transition in body-centred cubic tungsten. *J. Mater. Sci. Technol.* **106**, 33–40 (2022).

50. J. Wang, Z. Zeng, M. Wen, Q. Wang, D. Chen, Y. Zhang, P. Wang, H. Wang, Z. Zhang, S. X. Mao, T. Zhu, Anti-twinning in nanoscale tungsten. *Sci. Adv.* **6**, eaay2792 (2020).

51. J. Qian, C. Y. Wu, J. L. Fan, H. R. Gong, Effect of alloying elements on stacking fault energy and ductility of tungsten. *J. Alloys Compd.* **737**, 372–376 (2018).

52. A. Bisht, R. K. Koju, Y. Qi, J. Hickman, Y. Mishin, E. Rabkin, The impact of alloying on defect-free nanoparticles exhibiting softer but tougher behavior. *Nat. Commun.* **12**, 2515 (2021).

53. E. I. Preiß, B. Merle, M. Göken, Understanding the extremely low fracture toughness of freestanding gold thin films by in-situ bulge testing in an AFM. *Mater. Sci. Eng. A* **691**, 218–225 (2017).

54. P. Schweizer, P. Denninger, C. Dolle, E. Spiecker, Low energy nano diffraction (LEND) – A versatile diffraction technique in SEM. *Ultramicroscopy* **213**, 112956 (2020).

55. J. Holm, B. Caplins, J. Killgore, Obtaining diffraction patterns from annular dark-field STEM-in-SEM images: Towards a better understanding of image contrast. *Ultramicroscopy* **212**, 112972 (2020).

56. M. Gulde, S. Schweda, G. Storeck, M. Maiti, H. K. Yu, A. M. Wodtke, S. Schäfer, C. Ropers, Ultrafast low-energy electron diffraction in transmission resolves polymer/graphene superstructure dynamics. *Science* **345**, 200–204 (2014).

57. T. E. J. Edwards, Recent progress in the high-cycle fatigue behaviour of γ-TiAl alloys. *Mater. Sci. Technol.* **34**, 1919–1939 (2018).

58. J. Cho, Q. Li, H. Wang, Z. Fan, J. Li, S. Xue, K. S. N. Vikrant, H. Wang, T. B. Holland, A. K. Mukherjee, R. E. García, X. Zhang, High temperature deformability of ductile flash-sintered ceramics via in-situ compression. *Nat. Commun.* **9**, 2063 (2018).

59. S. Amini, M. Tadayon, S. Idapalapati, A. Miserez, The role of quasi-plasticity in the extreme contact damage tolerance of the stomatopod dactyl club. *Nat. Mater.* **14**, 943–950 (2015).

60. L. Chen, T. E. J. Edwards, F. Di Gioacchino, W. J. Clegg, F. P. E. Dunne, M.-S. Pham, Crystal plasticity analysis of deformation anisotropy of lamellar TiAl alloy: 3D microstructure-based modelling and in-situ micro-compression. *Int. J. Plast.* **119**, 344–360 (2019).

61. D. Wolf, V. Yamakov, S. R. Phillpot, A. Mukherjee, H. Gleiter, Deformation of nanocrystalline materials by molecular-dynamics simulation: Relationship to experiments? *Acta Mater.* **53**, 1–40 (2005).

62. Y. Guan, B. Chen, J. Zou, T. B. Britton, J. Jiang, F. P. E. Dunne, Crystal plasticity modelling and HR-DIC measurement of slip activation and strain localization in single and oligo-crystal Ni alloys under fatigue. *Int. J. Plast.* **8**, 70–88 (2017).

63. A. Guery, F. Hild, F. Latourte, S. Roux, Slip activities in polycrystals determined by coupling DIC measurements with crystal plasticity calculations. *Int. J. Plast.* **81**, 249–266 (2016).

64. A. J. Wilkinson, G. Meaden, D. J. Dingley, High-resolution elastic strain measurement from electron backscatter diffraction patterns: New levels of sensitivity. *Ultramicroscopy* **106**, 307–313 (2006).

65. T. E. J. Edwards, F. Di Gioacchino, A. J. Goodfellow, G. Mohanty, J. Wehrs, J. Michler, W. J. Clegg, Deformation of lamellar γ-TiAl below the general yield stress. *Acta Mater.* **163**, 122–139 (2019).

66. T. E. J. Edwards, F. Di Gioacchino, G. Mohanty, J. Wehrs, J. Michler, W. J. Clegg, Longitudinal twinning in a TiAl alloy at high temperature by in situ microcompression. *Acta Mater.* **148**, 202–215 (2018).

67. B. Horváth, R. Schäublin, Y. Dai, Flash electropolishing of TEM lamellas of irradiated tungsten. *Nucl. Instrum. Methods Phys. Res., Sect. B* **449**, 29–34 (2019).

68. D. M. Norfleet, D. M. Dimiduk, S. J. Polasik, M. D. Uchic, M. J. Mills, Dislocation structures and their relationship to strength in deformed nickel microcrystals. *Acta Mater.* **56**, 2988–3001 (2008).

69. Y. Meng, X. Ju, X. Yang, The measurement of the dislocation density using TEM. *Mater Charact* **175**, 111065 (2021).

70. P. Xiu, H. Bei, Y. Zhang, L. Wang, K. G. Field, STEM characterization of dislocation loops in irradiated FCC alloys. *J. Nucl. Mater.* **544**, 152658 (2021).

71. R. K. Ham, The determination of dislocation densities in thin films. *Philos. Mag.* **6**, 1183–1184 (1961).

72. T. E. J. Edwards, X. Maeder, J. Ast, L. Berger, J. Michler, in *Science Advances*. (Open Science Framework, 2022); DOI 10.17605/OSF.IO/U5A8W.

73. M. Negahban, *The Mechanical and Thermodynamical Theory of Plasticity* (CRC Press, 2012).

74. N. Rendler, I. Vigness, Hole-drilling strain-gage method of measuring residual stresses. *Exp. Mech.* **6**, 577–586 (1966).

75. G. B. Greenough, Strain measurement by x-ray diffraction methods. *Aeronaut. Q.* **1**, 211 (1949), 224.

76. M. T. Hutchings, A. D. Krawitz, *Measurement of Residual and Applied Stress Using Neutron Diffraction* (Springer Science & Business Media, 2012), vol. 216.

77. P. F. Rottmann, K. J. Hemker, Nanoscale elastic strain mapping of polycrystalline materials. *Mater. Res. Lett.* **6**, 249–254 (2018).

78. F. Hüe, M. Hÿtch, H. Bender, F. Houdellier, A. Claverie, Direct mapping of strain in a strained silicon transistor by high-resolution electron microscopy. *Phys. Rev. Lett.* **100**, 156602 (2008).

79. J. Jiang, T. B. Britton, A. J. Wilkinson, Evolution of dislocation density distributions in copper during tensile deformation. *Acta Mater.* **61**, 7227–7239 (2013).

80. H. Yu, J. Liu, P. Karamched, A. J. Wilkinson, F. Hofmann, Mapping the full lattice strain tensor of a single dislocation by high angular resolution transmission Kikuchi diffraction (HR-TKD). *Scr. Mater.* **164**, 36–41 (2019).

81. A. C. Leff, C. R. Weinberger, M. L. Taheri, Estimation of dislocation density from precession electron diffraction data using the Nye tensor. *Ultramicroscopy* **153**, 9–21 (2015).

82. M. A. Sutton, J. J. Orteu, H. Schreier, *Image Correlation for Shape, Motion and Deformation Measurements: Basic Concepts, Theory and Applications* (Springer Science & Business Media, 2009).

83. N. Lenoir, M. Bornert, J. Desrues, P. Bésuelle, G. Viggiani, Volumetric digital image correlation applied to x-ray microtomography images from triaxial compression tests on argillaceous rock. *Strain* **43**, 193–205 (2007).

84. F. Bourdin, J. C. Stinville, M. P. Echlin, P. G. Callahan, W. C. Lenthe, C. J. Torbet, D. Texier, F. Bridier, J. Cormier, P. Villechaise, T. M. Pollock, V. Valle, Measurements of plastic localization by heaviside-digital image correlation. *Acta Mater.* **157**, 307–325 (2018).

85. D. Breuer, W. Pantleon, U. Martin, U. Mühle, H. Oettel, O. Vöhringer, Modelling of the creep behaviour of a cobalt-based alloy by applying the kocks-mecking model. *Phys. Status Solidi A* **150**, 281–295 (1995).

86. C.-M. Kuo, C.-S. Lin, Static recovery activation energy of pure copper at room temperature. *Scr. Mater.* **57**, 667–670 (2007).

87. C. R. Weinberger, G. J. Tucker, S. M. Foiles, Peierls potential of screw dislocations in bcc transition metals: Predictions from density functional theory. *Phys. Rev. B* **87**, 054114 (2013).

88. K. S. Havner, *Finite Plastic Deformation of Crystalline Solids* (Cambridge Monographs on Mechanics, Cambridge Univ. Press, 1992).

89. A. Ma, F. Roters, A constitutive model for fcc single crystals based on dislocation densities and its application to uniaxial compression of aluminium single crystals. *Acta Mater.* **52**, 3603–3612 (2004).

90. S. G. Roberts, M. Ellis, P. B. Hirsch, in *Fundamental Aspects of Dislocation Interactions*, G. Kostorz, H. A. Calderon, J. L. Martin, Eds. (Elsevier, 1993), pp. 135–140.

91. M. Lefebvre, R. K. Keeler, R. Sobie, J. White, Propagation of errors for matrix inversion. *Nucl. Instrum. Methods Phys. Res. A* **451**, 520–528 (2000).
